# Topical treatment of vaginal dryness with a non-hormonal cream in women undergoing breast cancer treatment - An open prospective multicenter study

**DOI:** 10.1371/journal.pone.0210967

**Published:** 2019-01-24

**Authors:** Dimitrios Chatsiproios, Iris M. Schmidts-Winkler, Lisa König, Clarissa Masur, Christoph Abels

**Affiliations:** 1 Gynaecologicum, Kreuzlingen, Switzerland; 2 DR. AUGUST WOLFF GmbH & CO. KG–ARZNEIMITTEL, Bielefeld, Germany; Rabin Medical Center, ISRAEL

## Abstract

This open, prospective, multicenter, observational study was performed to investigate the efficacy and safety of a non-hormonal cream in women undergoing breast cancer treatment and experiencing vulvovaginal dryness symptoms. Overall, 128 patients from 22 study centers participated. The cream was applied to the vagina and vulva for 28 days. For the efficacy analysis, changes in subjective symptoms (feeling of dryness, itching, burning, pain independent of sexual intercourse, dyspareunia, urinary incontinence) were evaluated. Additionally, the following objective diagnostic findings were assessed by a physician: thinning of vaginal epithelium, redness, petechiae, and discharge. Safety and tolerability were assessed by evaluating type and frequency of adverse events, including adverse medical device-related effects. The frequency and intensity of all subjective symptoms significantly improved from baseline at 28 days (p<0.0001). Additionally, 21.4% of patients were completely free of symptoms (p<0.0001) and urinary incontinence was improved or eliminated in 30.8% of women. The overall sum score for all four objective findings was significantly improved from baseline at 28 days (p<0.0001). The frequency of petechial bleedings was significantly reduced (p<0.0001). Further, significant decreases in the severity of vaginal epithelium thinning, redness and petechiae were observed (p<0.0001). More than 88% of patients and investigators assessed the efficacy and tolerability as being good or very good. No serious adverse events were documented. This study demonstrates that the investigated cream is an effective and safe non-hormonal, topical option in the treatment of vulvovaginal dryness symptoms in patients undergoing breast cancer treatment for. However, the study duration and follow-up time of 4 weeks as well as the non-randomized trial design are limitations of the study.

## Introduction

Induction of premature menopause by chemotherapy and the increased use of aromatase inhibitors leading to estrogen deprivation have resulted in a high prevalence of severe side effects in breast cancer patients [1–5]. Vulvovaginal atrophy (VVA), also termed *genitourinary syndrome of menopause* (GSM), depicts in this context one of the long-term side effects [6]. The main symptom vulvovaginal dryness (VVD) negatively affects the quality of life and leads to sexual problems and dysfunctions [3, 5].

VVD symptoms develop after starting treatment in a high percentage of breast cancer patients [7], which constitute one of the main reasons for discontinuation of and non-adherence to endocrine treatment [8, 9], accounting for a decreased survival [10]. Medical interventions improving quality of life and therefore resulting in an enhanced compliance to endocrine hormone therapy may thus be critical for improving breast cancer survival rates.

Women experiencing VVD symptoms suffer from discomfort in daily life, including feelings of dryness, itching, burning, and pain, as well as painful sexual intercourse [1, 11, 12]. On examination, the vaginal epithelium appears thin and dry, with areas of both erythema and pallor. The vaginal lining may exhibit petechiae [13, 14].

Systemically or vaginally administered estrogen is one therapeutic option for moderate-to-severe VVA symptoms in healthy postmenopausal women. However, it could potentially raise the risk of breast cancer recurrence, especially in patients with hormone-receptor-positive tumors. Therefore, systemic administration is in principle contraindicated in these patients [15, 16]. With regard to local estrogen treatment, there are currently still insufficient data to confirm its safety in women with breast cancer [14]. Thus, non-hormonal interventions are recommended as first-line treatment for women with VVD symptoms [14], especially in women with a history of hormone-dependent breast cancer [17, 18].

There are several non-hormonal treatment options for VVD symptoms, but the majority of these lubricants or moisturizers are water-based gels, that consist of a combination of a thickening agent in a water-soluble base and non-hormonal substances [19]. In contrast, an oil-in-water emulsion (cream) supplies not only water to moisturize and increase hydration, but also lipids acting as a lubricating film on the epithelium to prevent loss of water. Lubricants are intended to be used immediately before sexual activity, and there is no evidence of any long-term, vaginal therapeutic effect. In contrast, vaginal moisturizers exert their effects by replacing normal vaginal secretions, are applied internally and externally at regular intervals, long-lasting and can effectively decrease vaginal dryness [20, 21].

Overall, women undergoing breast cancer treatment exhibit symptoms of VVD but have less therapeutic options than healthy postmenopausal women because local estrogen treatment is avoided by cancer patients and physicians due to insufficient data regarding safety [14].

Therefore, the aim of this prospective, observational, multicenter study was to evaluate the efficacy and tolerability of a non-hormonal, moisturizing cream in this specific patient population.

## Materials and methods

### Study design

This prospective, multicenter, open-label, non-interventional, observational study was conducted at 22 centers in Germany from January to July 2010. Female breast cancer patients aged ≥18 years undergoing chemotherapy or endocrine treatment (and up to three months after completion of anticancer treatment) and suffering from VVD were selected for study participation at the different study centers. The study protocol was approved by the Freiburg Ethics Commission International (code 09/2526) and written informed consent was obtained from each participant. The study has been registered at ClinicalTrials.gov (NCT02269826). Women who had VVD symptoms before starting anticancer treatment as well as women who were undergoing radiotherapy, had vaginal infections or other vaginal or vulvar disorders, were currently using products for VVD or VVA, had known allergies to the ingredients of the study product, or who were pregnant or breastfeeding were excluded. The study product, an oil-in-water emulsion (Vagisan Moisturising Cream, Dr. August Wolff GmbH &Co. KG Arzneimittel, Germany) contains 23% lipids and is adjusted to a pH of 4.5 with lactic acid to be consistent with the pH of a healthy vaginal environment. The osmolality of the cream is 374 mosmol/kg and thus in accordance with the WHO-recommended 380 mosmol/kg for personal lubricants [22, 23]. The study product does not contain estrogen and therefore, systemically estrogen level changes were not expected and investigated. This medical device was administered by the participants themselves for 28 days. Efficacy and safety data were assessed at baseline (V1) and day 28 (V2). Patients were free to withdraw from the study at any time and for any reason.

The dosage and treatment schedule of the cream was in accordance with the instructions in the package leaflet: the recommended initial intravaginal application is 2.5 g of cream per day in the evening (first week) and from weeks 2 to 4 daily or as needed; the recommended vulvar application is 0.5 g of cream (one fingertip unit) once daily or several times per day, according to individual needs.

### Efficacy and safety assessments

At baseline, demographics, medical history, concomitant diseases and corresponding medications (especially regarding tumor diagnosis and oncological therapies) were recorded.

At each visit, the patient documented subjective symptoms, such as feelings of dryness, itching, burning, as well as pain independent of sexual intercourse and rated the symptom intensity (0 = none, 1 = hardly pronounced, 2 = moderately pronounced, 3 = quite pronounced, 4 = very pronounced). The intra-individual difference in sum scores of these four subjective symptoms (values thus ranging from 0 to 16) between V1 and V2 was also calculated. In addition, patients were questioned about the existence and intensity of dyspareunia, using the same rating scale as for the subjective symptoms. Changes regarding urinary incontinence at baseline and at 28 days (improvement, no change, deterioration) were documented.

At each visit, a colposcopic examination was conducted and the following objective diagnostic findings were evaluated and documented in the physician’s case report form: thinning of the vaginal epithelium, redness, petechial bleedings, and discharge. Severity of the findings were rated by using a scale which ranged from 0 to 4 (0 = none, 1 = hardly pronounced, 2 = moderately pronounced, 3 = quite pronounced, 4 = very pronounced). Additionally, the intra-individual difference of the sum scores of objective findings between baseline and day 28 was calculated. The sum score was the sum of the severity scores for the four objective findings and represents integer values ranging from 0 to 16. The intra-individual difference of the sum scores is thereby calculated by the difference of the sum scores at V1 and V2.

Safety and tolerability were assessed by evaluating the type and frequency of adverse events (AEs) and adverse medical device events (AMDEs). Tolerability was analyzed by asking the patients about the incidence of predefined, well-known side effects of the cream, using the following defined parameters: “transient itching or burning (less than 10 minutes)”, “longer-lasting itching or burning (10–60 minutes)”, “severe intolerance (itching, burning, swelling more than 60 minutes)”, “discharge of the cream”. Moreover, the patients were questioned about any AEs, with a subsequent causality assessment by the investigator. At day 28, tolerability was evaluated by the patient and the physician (“very good”, “good”, “satisfactory”, “sufficient”, “poor”, or “unsatisfactory”).

At day 28, further concomitant conditions developing within the last four weeks and their corresponding medications as well as the frequency of intravaginal and vulvar applications of the cream were recorded. Additionally, both the patient and the physician evaluated the effectiveness of the study product (“very good”, “good”, “satisfactory”, “sufficient”, “poor”, “unsatisfactory”).

## Statistical analysis

SAS software (Version 9.1.3, Heidelberg, Germany) was used in this study for data collection, management and statistical analysis. A total number of at least 100 participants were needed for obtaining valid results regarding the change of subjective symptoms. A statistical analysis plan was defined prior to study start. Descriptive statistical methods like frequency counts and summary statistics, including arithmetic mean ± standard deviation (SD), median and range, were used. Frequency distributions for discrete variables were provided as percentage in relation to the total sample. Statistical significance was determined by using an alpha level of 0.05 and two-sided tests. Quantitative variables were compared using the chi-square test. Evaluation of parameter changes during the study course were performed by intra-individual difference analysis (V1 vs. V2) using the Wilcoxon signed-rank test.

## Results

### Study population

**Fig 1:**
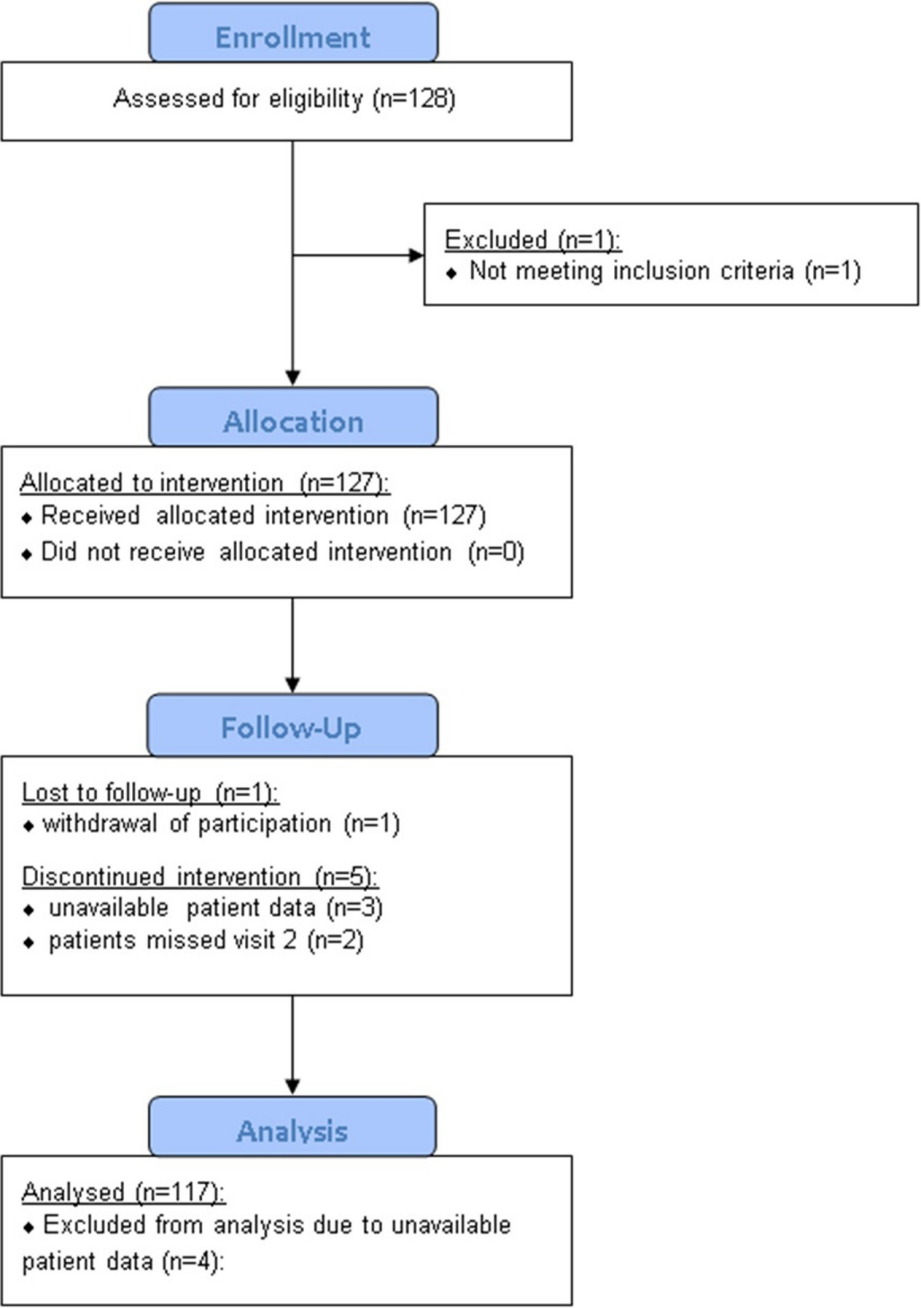
Patient flow chart.

128 patients from 22 centers were enrolled. Due to reasons unrelated to treatment, 11 subjects were either excluded from the analysis (n = 6; 4.7%) or withdrew prematurely (n = 5; 3.9%). In total, 117 patients from 22 centers completed the study and were available for evaluation (Fig 1)

The mean study duration was 32 ± 6.1 days (median: 31 days; range 15–55 days). Of the 117 subjects, eight applied the cream intravaginally only (median: 6.5 times per week; range: 1–7 times), nine applied the study product on the vulvar area only (median: 5.0 times per week; range: 2–7 times) and 100 patients applied the study product both intravaginally (median: 3.0 times per week; range: 1–7 times) and on the vulvar region (median: 5.0 times per week; range: 1–7 times).

Demographic and baseline characteristics are summarized in Table 1. Nearly all patients had been diagnosed with breast cancer (n = 116; 99.1%), except one patient with endometrial cancer. This patient also suffered from VVD symptoms due to anticancer treatment and therefore was not excluded. As shown in Table 1, patients in the study population were undergoing different anticancer treatments.

**Table 1:** Demographic and baseline characteristics.

**Variable**	**n**	
Age [years]		
Mean (SD)	52 (9.9)	
Median (Range)	51 (30–85)	
**Baseline characteristics**	**n**	**%**
Tumor diagnosis		
Breast cancer	116	99.1
Endometrial cancer	1	0.9
Anticancer Treatment		
Surgery + Chemotherapy + Antiestrogens	33	28.2
Surgery + Chemotherapy + Aromatase Inhibitors	26	22.2
Surgery + Chemotherapy	18	15.4
Surgery + Antiestrogens	15	12.8
Other combinations (5 different combinations)*	25	21.4
Total	117	100.0

* n ≤ 8 for each combination

### Efficacy

**Fig 2:**
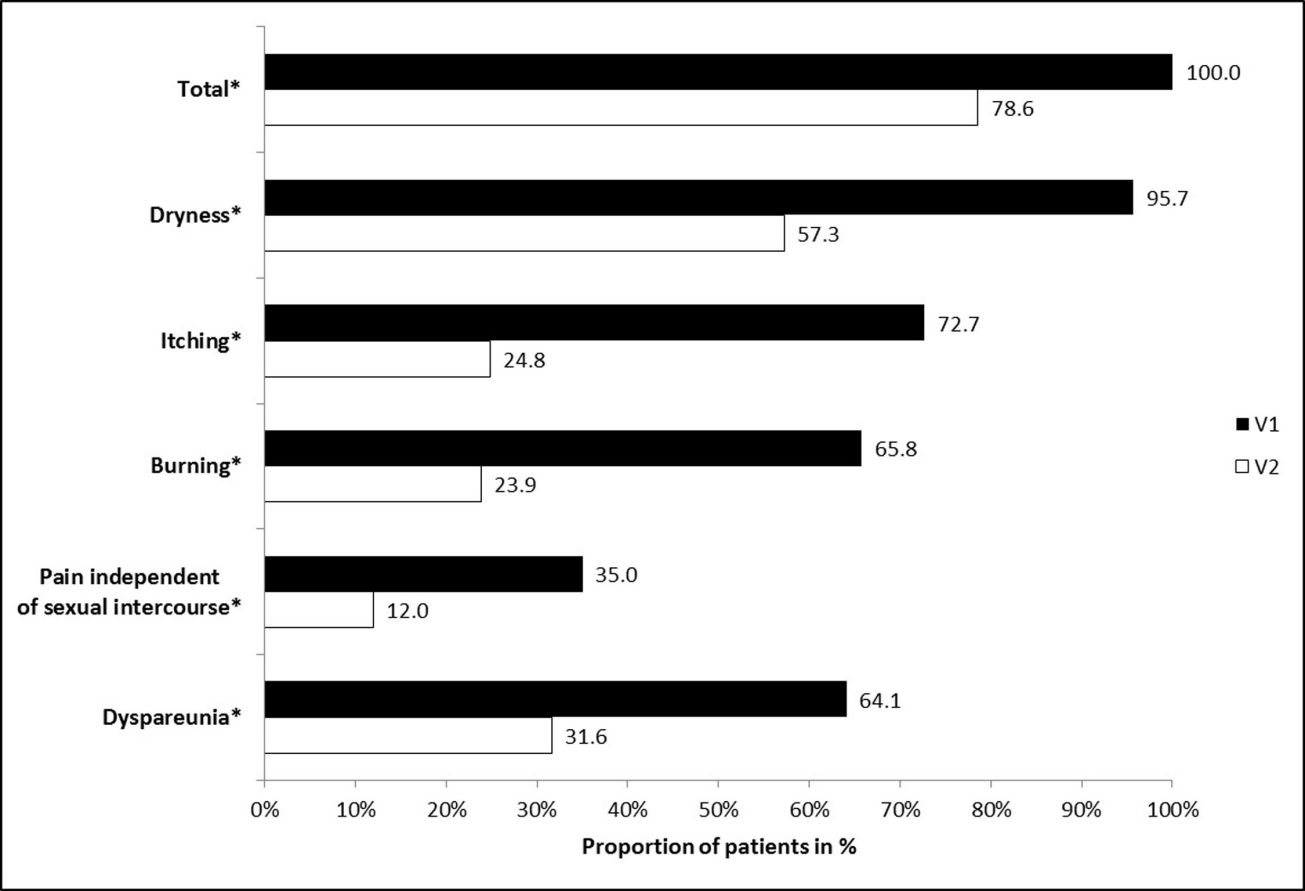
Proportion of patients with subjective symptoms of vaginal dryness during the study period. All 117 patients reported symptoms of vaginal dryness at the beginning of the study and were further analyzed for each subjective symptom during the study period. * Chi-square test: p<0.0001; V1 = Visit 1 at baseline, V2 = Visit 2 on day 28.

At baseline (V1), symptoms related to “feeling of dryness” were reported by 112 patients (95.7%), “burning” by 77 patients (65.8%), “itching” by 85 patients (72.6%) and “pain independent of sexual intercourse” by 41 patients (35.0%). The frequencies of these symptoms (Fig 2; p<0.0001) were statistically significantly reduced at day 28 (V2), compared to baseline. Based on the individual symptom results, a highly positive effect was observed for the intra-individual difference of the sum score of subjective symptoms (sum scores at V1 –sum scores at V2; p<0.0001). Average value of intra-individual differences in the total sum scores of the subjective symptoms was 3.81 (with SD of 3.06; median of 3, minimum -4, maximum 13). At V2, 21.4% of patients (n = 25) were completely free from subjective symptoms (Fig 2; p<0.0001).

Of the women having sexual intercourse (n = 89), 75 (84.3%) reported experiencing dyspareunia at the start of the study. Frequency of dyspareunia was significantly decreased at day 28 (Fig 2; p<0.0001) and the intensity of this symptom was substantially improved as well.

With respect to urinary incontinence, 30.8% (n = 8) of the 26 women experienced improvement (n = 5; 19.2%) or full elimination (n = 3; 11.5%) by the end of the study (S1 Fig).

**Fig 3:**
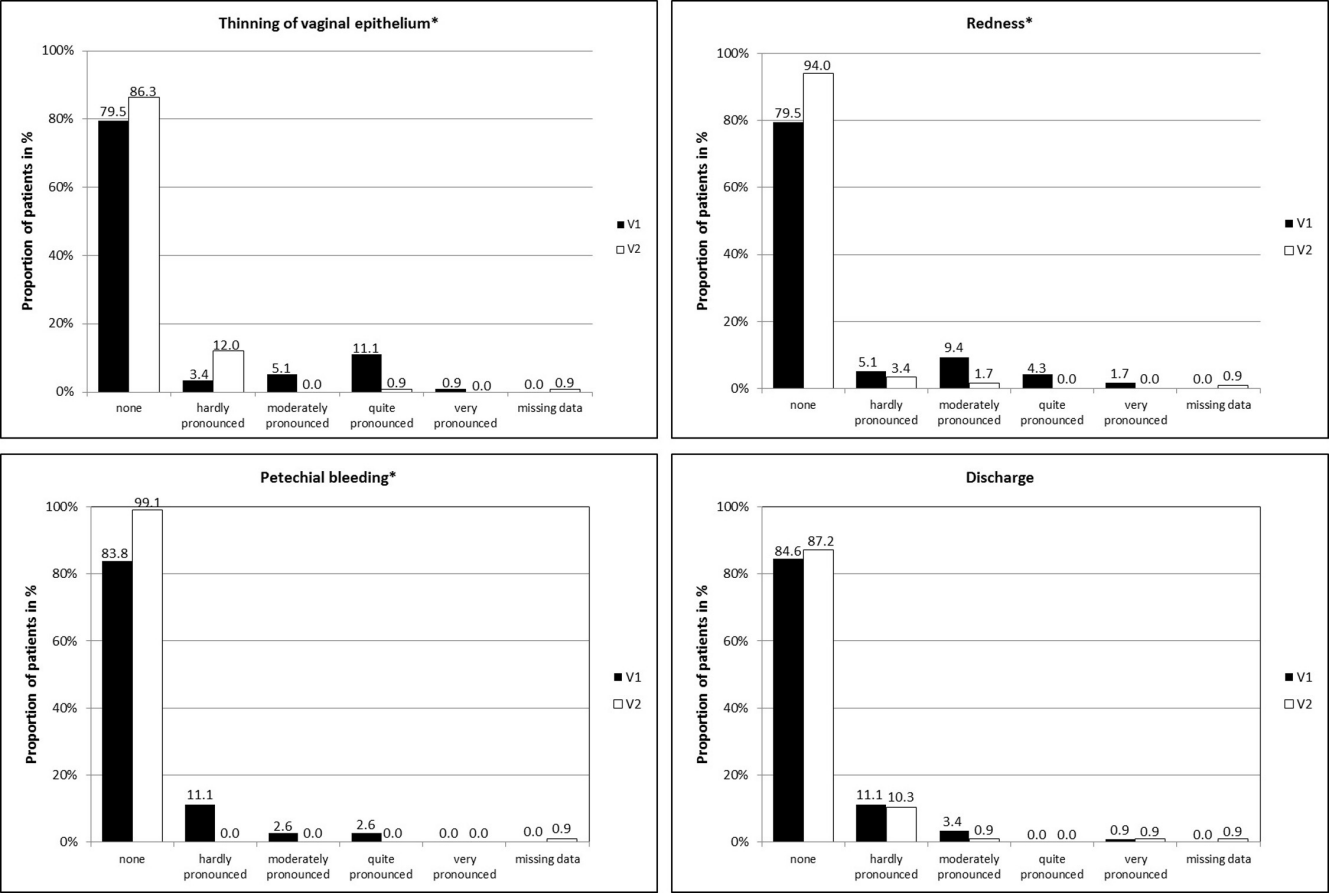
Severity of objective findings during the study period. All 117 patients underwent the gynecological examination at study start, one patient missed the examination at the end of the study (n = 116). Due to rounding of the percentages, the total is not 100%. * Wilcoxon signed-rank test: p<0.0001; V1 = Visit 1 at baseline, V2 = Visit 2 on day 28.

Objective findings were present in only 24 patients at study start. Colposcopy revealed thinning of the vaginal epithelium in 24 of 117 patients (20.5%), redness in 24 (20.5%), petechial bleeding in 19 (16.2%) and discharge in 5 patients (4.3%). By the end of the 28-day treatment, the number of patients with objective findings decreased markedly and all patients were free of petechial bleedings (p<0.0001). The severity of three of the objective findings (thinning of the vaginal epithelium, redness and petechial bleedings) was highly significantly decreased (Fig 3; p<0.0001) compared with baseline values whereas only a trend towards reduction in discharge was observed (p = 0.2402). The overall sum score for all four objective findings was highly significantly improved from baseline at 28 days (p<0.0001). Average value of intra-individual differences in the total sum scores of the objective findings was 1.03 (with SD of 2.47; median of 0.00, minimum -2, maximum 11).

**Fig 4:**
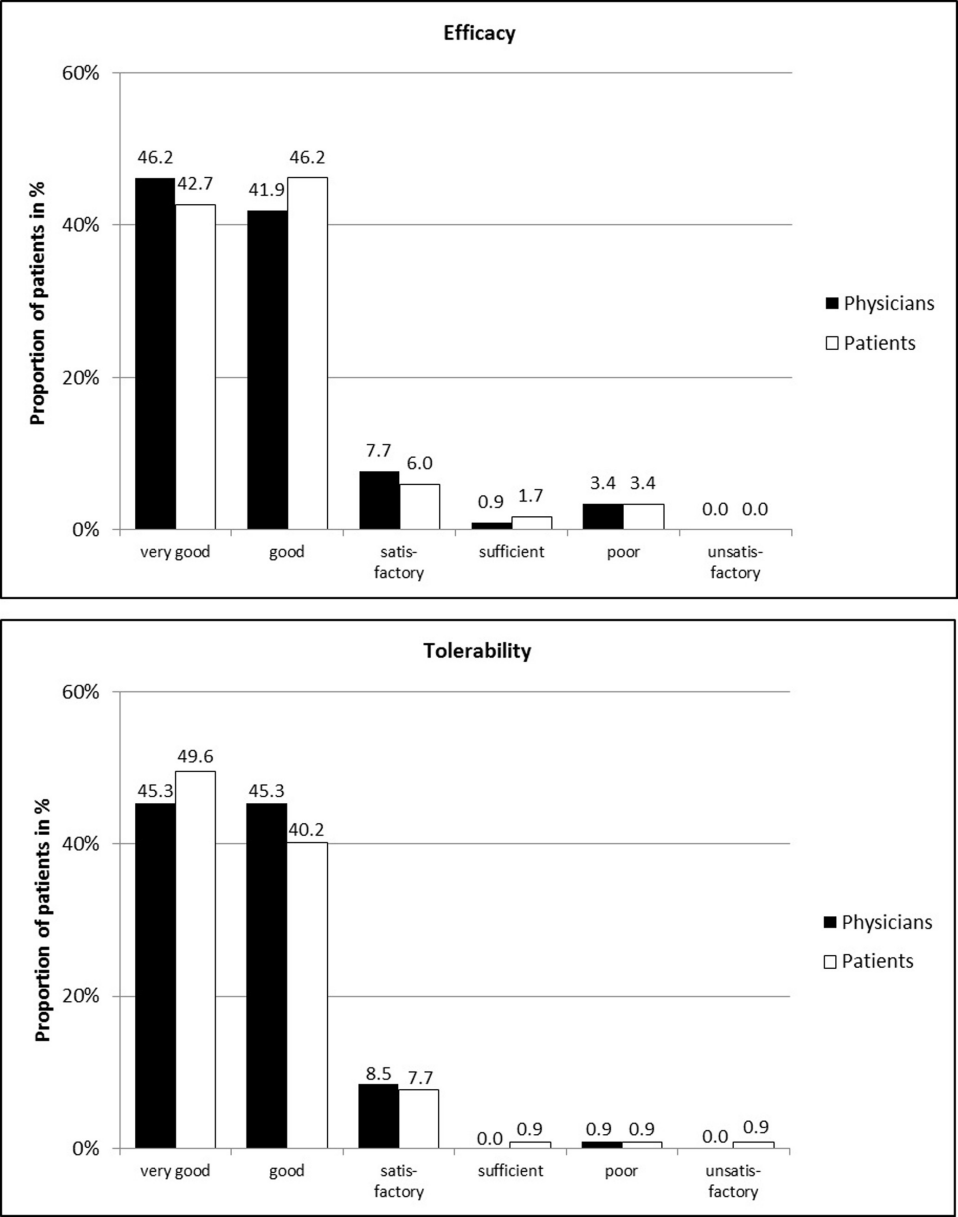
Physicians’ and patients’ assessments of efficacy and tolerability. Overall, all 117 patients and their physicians assessed the efficacy and tolerability. Due to rounding of the percentages, the total is not 100%.

Physicians assessed the efficacy of the study product as being “very good” or “good” for 88.1% of patients (n = 103). Similarly, 88.9% of the patients (n = 104) assessed the efficacy of the cream as being “very good” or “good” (Fig 4).

### Safety

When asked about the incidence of predefined, well-known side effects of the cream, transient itching or burning (shorter than 10 minutes) was reported by 19 patients (16.2%), and longer-lasting itching or burning (10 to 60 minutes) by another 4 patients (3.4%). No patient experienced severe intolerance (itching, burning or swelling for more than 60 minutes). Cream discharge was reported by 27.4% of patients (n = 32).

Thirteen patients (11.1%) reported 20 AEs. Four women (3.4%) experienced five AEs assessed as AMDEs by the investigator. All of these AMDEs were of a mild (2 out of 5; 40%) or moderate (3 out of 5; 60%) intensity. Nine (7.7%) patients reported 15 AEs not considered related to the study product (Table 2). In 104 patients (88.9%) no AE was observed. No serious AEs occurred during the course of the study.

**Table 2:** Adverse events (AE) and causality assessment (n = 117).

Adverse Events (AE)		n	%
No AE		104	88.9
AE without causality to the medical device (no AMDE*)		9	7.7
AE with causality to the medical device (AMDE*)	Deterioration of pain independent of sexual intercourse	2	1.7
	Deterioration of dryness and pain independent of sexual intercourse	1	0.85
	Deterioration of itching	1	0.85

* Adverse medical device event

Physicians described the tolerability as being “very good” or “good” for 90.6% of patients (n = 106). The patients’ assessments revealed similar results: 89.7% (n = 105) of patients assessed the tolerability of the cream as being “very good” or “good” (Fig 4).

## Discussion

The present study was an open, observational study performed over a period of 4 weeks in 117 patients, reflecting the everyday life of breast cancer patients with VVD symptoms and their individual needs regarding frequency of product application. Here, this non-hormonal cream significantly reduced subjective symptoms and even improved objective findings of VVD in women undergoing breast cancer treatment. The results confirm the findings in post-menopausal women, in which efficacy has already been demonstrated in a previous study [24]. Notably, more than 20% of patients in this study reported being free of symptoms after the treatment period. In addition, about one third of women experienced improvement or full elimination of urinary incontinence after using this non-hormonal cream for 28 days. Moreover, application of the product resulted in a highly significant reduction regarding severity of objective findings of vaginal dryness, such as thinning of the vaginal epithelium, redness and petechial bleedings, and at day 28, all patients were free of petechial bleedings. Apart from transient sensations of burning and itching after product application there were no AMDEs. Physicians and patients assessed the efficacy and tolerability of the cream in the majority of cases as being “good” or “very good”. No serious adverse effects were reported.

So far, only a few studies assessed efficacy of non-hormonal vaginal products for VVD in breast cancer patients, all of them using water-based gels [25–27]. The present study is the first one investigating a non-hormonal cream (oil-in-water emulsion) for breast cancer therapy-related VVD. The superiority of a cream compared to a gel formulation has already been shown in a previous study in pre-, peri- and postmenopausal patients with VVD symptoms [24], confirming the benefit of lipid-containing preparations in the treatment of skin dryness symptoms associated with VVD. Oil-in-water emulsions (creams) supply water to moisturize and increase hydration and, provide lipids that act as a lubricating film on the epithelium to prevent water loss from the upper skin layers and replace skin surface lipids. This treatment principle takes into account the fact that vulvar and vaginal skin are not mucosal structures but consist of a keratinized and non-keratinized stratified epithelium, respectively.

Other breast cancer treatment-related studies include a double-blind crossover study that compared a polycarbophil-based gel (Replens) with a water-soluble lubricating placebo in 45 patients [27] or a parallel-group study that compared two low-dose vaginal estrogen treatments (estriol cream 0.25 mg, 10 patients; estradiol tablets 12.5 μg, 8 patients) with polycarbophil-based gel (8 patients) [25]. Another randomized study compared a lactic acid-containing pH-balanced gel (pH 4.0) with placebo (a gel without lactic acid and a pH of 7.2) in 86 patients [26]. Duration of treatment periods varied from four [27] to twelve weeks [25, 26]. The study populations in the prior mentioned studies were comparable to this study, being defined as breast cancer patients currently receiving antineoplastic treatment (chemotherapy and/or endocrine cancer treatment) and having persistent vaginal dryness (with pain) and/or itching [25–27]. Only one study compared the efficacy of a vaginal estradiol treatment or a vaginal moisturizer versus a placebo gel/tablet regarding symptom severity of vaginal dryness, however, in a cohort of postmenopausal women [28].

Regarding subjective symptoms, feeling of dryness [24–30], itching [24, 25, 27–31], burning/ irritation [24, 25, 28, 31] as well as dyspareunia [24–31] and urinary incontinence [25, 29] were commonly assessed in these studies. However, because validated questionnaires are lacking, selection of and scoring systems for VVD symptoms vary, which makes comparisons between studies difficult. In this study, all subjective symptoms (using a score from 0 to 4 for each symptom) improved significantly as compared to baseline, both regarding frequency and intensity. In the study comparing the polycarbophil-based gel with placebo (using a score from 0 to 4), vaginal dryness symptoms decreased by 64% and 62%, and dyspareunia decreased by 60% and 41%, respectively (average scores), as compared to baseline; there seemed to be no substantial impact of either of the products on itching [27]. In the study comparing Replens with local estrogens, vaginal symptoms were also scored from 0 to 4, and were combined to produce a Vaginal Symptoms Score (VSS). The VSS decreased significantly after the first 4 weeks in the Replens-arm compared with baseline, although this decrease was less pronounced than the one observed with the local estrogen treatments. However, the VSS returned to baseline values at 8 weeks post-Replens treatment [25]. In a placebo-controlled study of a pH-balanced gel, dryness and dyspareunia were assessed with a visual analog scale that ranged from 0 to 10.0. Vaginal dryness and dyspareunia improved more in the pH-balanced gel group than in the placebo group [26]. In contrast, Mitchell *et al*. reported that neither a vaginal estradiol tablet nor a vaginal moisturizer had a benefit over a placebo vaginal tablet/gel in reducing postmenopausal vulvovaginal symptoms [28].

The evaluation of objective VVD signs in published studies has been inconsistent as well. In this study, objective signs such as thinning of the vaginal epithelium, redness, petechial bleedings, and discharge were assessed, using a score from 0 to 4 for each finding. At day 28, severity of thinning of the vaginal epithelium, redness, and petechial bleedings were highly significantly reduced. In the study comparing the polycarbophil-based gel with placebo, vaginal elasticity, secretions, mucosal integrity, and moisture were only assessed at baseline, using a scale of 1 to 5; however, no data were shown at all for the end of the study [27]. In the study comparing polycarbophil-based gel with local estrogen products, a wider range of estrogen-influenced parameters were evaluated, such as the Vaginal Health Index (VHI; evaluation of the vaginal mucosa appearance), and the Karyopyknotic Index (KI); no significant changes in VHI and KI were recorded for the vaginal moisturizer [25]. Vaginal maturation index (VMI) and VHI were also assessed in the study which compared the pH-balanced vaginal gel with placebo; both VMI and VHI were improved by both products, with a significant difference in favor of the pH-balanced gel [26].

Transient sensations of burning and itching after application of the cream occurred in 19.6% of patients overall. This is a slightly higher rate when compared to the previous study of this cream in women of all ages suffering from VVD symptoms due to causes other than breast cancer treatment [24]; in this study, 15% of patients using the cream (and 32% of patients using a vaginal gel) reported these side effects. These adverse effects can generally be separated by their acute nature compared with the more chronic nature of the VVD symptoms and occur at a similar rate with other products. In a study with breast cancer patients comparing polycarbophil-based gel with placebo, 16.7% complained of side effects (local burning, irritation, itching or discharge) at least once while using polycarbophil-based gel and 20% while using placebo [27], rates that are comparable to the rates of side effects in this study. In a trial comparing a vaginal pH-balanced gel versus placebo in women undergoing breast cancer treatment, the most common adverse effects were irritation or burning sensation (36.7% and 16.3%, respectively) as well as itching (26.5% and 6.1%, respectively) [26]. The rates of adverse effects associated with the pH-balanced gel were considerably higher than those observed with the cream investigated in the previous [24] and current study.

At the beginning of the study, 22.2% of the patients reported urinary incontinence. Overall, the prevalence of urinary problems during breast cancer treatment was reported differently and occurs in up to 55% of patients undergoing breast cancer treatment [2, 32–35]. However, the type of urinary problem in these studies was not clearly defined, in comparison to our study focusing particularly on urinary incontinence. In contrast to Morales *et al*., the patients experiencing urinary incontinence at baseline in our study reported a significant improvement during treatment [35]. The beneficial effect of a non-hormonal moisturizing cream regarding urinary incontinence has not been reported in breast cancer patients before. However, non-hormonal treatment of VVD symptoms improves the quality of life in postmenopausal women [21], which may have a beneficial effect beyond symptoms of vulvovaginal dryness [34, 36].

### Limitations of the study

As this was an open, observational study, no strict primary endpoint was defined, however, the assessment of the subjective symptoms (dryness, burning, itching and pain independent from sexual intercourse) was considered to be most important. Efficacy parameters evaluated these subjective symptoms and objective signs (thinning of the vaginal epithelium, redness, petechial bleedings and discharge) in accordance with other studies on VVD. Further, the study duration of 28 days does not simulate a long-term use of the cream in cancer patients. Additional data on a long-term use regarding efficacy and tolerability have to be gathered in this special patient group. Besides focusing on the treatment of VVD symptoms in breast cancer patients, one of 117 patients suffering from endometrial cancer and anti-cancer treatment associated VVD symptoms was included in this study. The most important outcome of this study–the change of subjective VVD symptoms—is negligibly influenced by including this endometrial cancer patient, as all 117 cancer patients suffer from VVD. The delay in publication of this study was caused by company internal reasons.

## Conclusion

In conclusion, Vagisan Moisturising Cream is an effective and safe non-hormonal option for the treatment of VVD symptoms, not only in postmenopausal women but also in women undergoing cancer treatment. Since long-term data on the safety of local estrogen treatment is still lacking it is important to have an effective and safe non-hormonal option, especially for breast cancer survivors with estrogen-dependent tumors. Further experiences regarding long-term use (> 4 weeks of follow up) of the investigated cream must be gathered in a randomized trial.

## Supporting information

S1 Fig Proportion of patients with urinary incontinence and the symptomatic change during treatment. At study start 26 of 117 patients reported urinary incontinence. The patients having urinary complaints were questioned again after the study course regarding the change of urinary incontinence.
(TIF)

S1 File TREND checklist. (PDF)

S2 File Observationplan in English. (PDF)

S3 File Observationplan in German. (PDF)

## Abbreviations

AE: Adverse event
AMDE: Adverse medical device event
KI: Karyopyknotic Index
VHI: Vaginal Health Index
VMI: Vaginal Maturation Index
VVA: Vulvovaginal atrophy
VVD: Vulvovaginal dryness
VSS: Vaginal Symptoms Score
