# Supplementary figures and images for: Topical treatment of vaginal dryness with a non-hormonal cream in women undergoing breast cancer treatment - An open prospective multicenter study
Source: PLoS One. 2019 Jan 24;14(1):e0210967. doi: 10.1371/journal.pone.0210967 (PMC6345451; doi:10.1371/journal.pone.0210967)

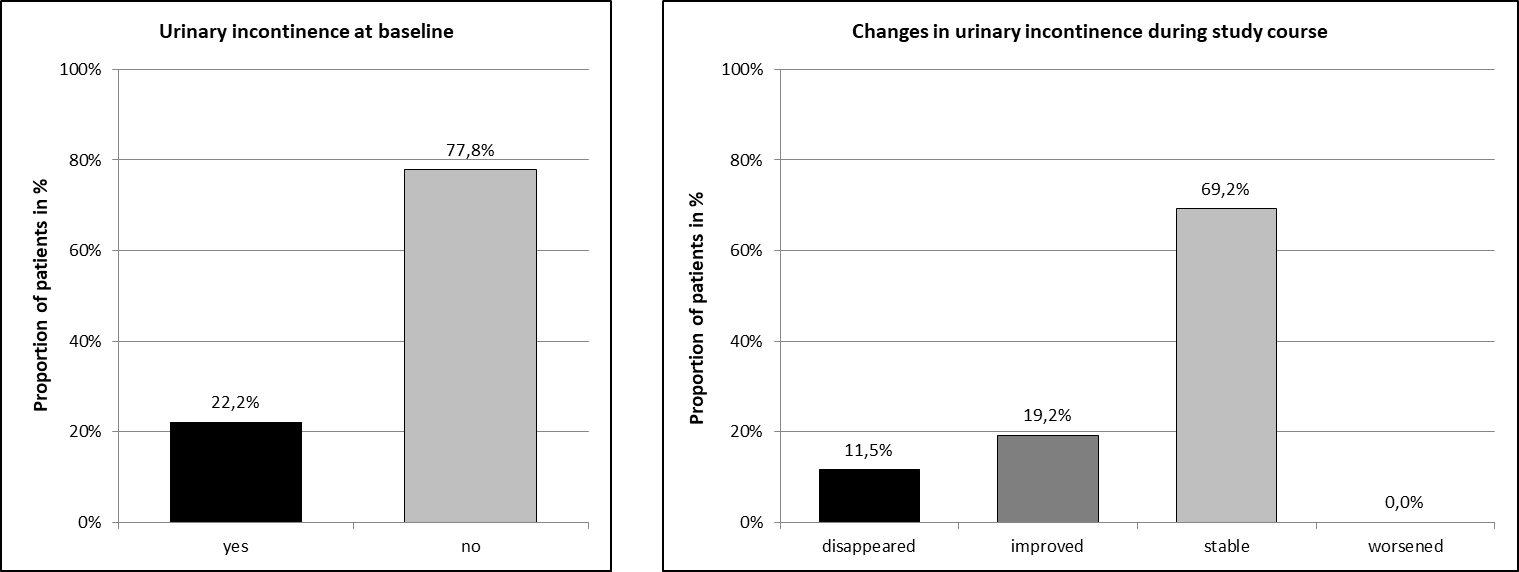

Supplement: S1 Fig — At study start 26 of 117 patients reported urinary incontinence. The patients having urinary complaints were questioned again after the study course regarding the change of urinary incontinence. (TIF) [file pone.0210967.s001.tif]
