# Supplementary material for: Topical treatment of vaginal dryness with a non-hormonal cream in women undergoing breast cancer treatment - An open prospective multicenter study
Source: PLoS One. 2019 Jan 24;14(1):e0210967. doi: 10.1371/journal.pone.0210967 (PMC6345451; doi:10.1371/journal.pone.0210967)
Supplement: S2 File — (PDF) [file pone.0210967.s003.pdf]

Multicentric Study
on the application of Vagisan® Moisturising Cream in breast cancer patients suffering from the symptoms of vulvovaginal dryness

OBSERVATION PLAN

The information contained in this document is **strictly confidential** and may not be shared with unauthorised individuals.

Table of Content

1. ADDRESSES AND RESPONSIBILITIES.. 1

2. SYNOPSIS 3

3. INTRODUCTION 7

4. OBJECTIVE OF THE APPLICATION STUDY 11

5. QUALITY ASSURANCE 12

6. OBSERVATION SCOPE / ORGANIZATION 13

7. OBSERVATION PROCEDURE 15

8. DEFINITIONS FOR MEDICAL DEVICE SAFETY...18

9. REPORT OR DOCUMENTATION OF SAE, ADVERSE MEDICAL DEVICE EFFECTS (AMDE), INCLUDING SUSPECTED ADVERSE MEDICAL DEVICE EFFECTS............21

10. DATA MANAGEMENT AND BIOMETRIC ANALYSIS 22

11. DUTY OF NOTIFICATION...23

12. BIBLIOGRAPHY...24

13. SIGNATURES 25

1. Addresses and responsibilities

**Scientific supervisor**	
**Medical device safety officer**	
**Sponsor's medical expert in gynaecology**	
**Biometry and Project Manager, Reporting**	
**Monitoring**	

Addresses and responsibilities (continued)

**Sponsor**	
**Sponsor's Project Manager**	

2. Synopsis

Study title	Multicentric study on the application of Vagisan® Moisturising Cream in breast cancer patients suffering from the symptoms of vulvovaginal dryness
Objective of the study	To investigate the suitability of Vagisan® Moisturising Cream in breast cancer patients with the objective of determining whether an improvement of the subjective symptoms and the objective findings of vulvovaginal dryness can be achieved with good tolerability
Target group	Breast cancer patients suffering from the symptoms of vulvovaginal dryness (frequent adverse side effectduring or after oncological treatment, such as chemotherapy or other medication, for example, aromatase inhibitors or anti-oestrogens)
Study parameters	Tolerability<br>- Type and frequency of side effects (adverse medical device effects , AMDEs)<br>- General assessment of tolerability (doctor / patient)<br>Efficacy<br>- Subjective symptoms (feeling of dryness; itching; burning; pain independent of intercourse; pain during intercourse, incontinence)<br>- Objective findings (thinning of the vaginal epithelium, redness; petechial bleeding, discharge)<br>- General assessment of efficacy (doctor / patient)<br>Overall assessment of the medical device<br>- Comprehensive evaluation of Vagisan® Moisturising Cream medical device (doctor / patient)
Study design	Multicentric application study of a medical device
Centres	Gynaecology clinics
Number of centres	About 30 clinics with at least 5 patients at each clinic
Number of patients	N = 150
Inclusion criteria	- Women over the age of 18<br>- Pre-menopausal women with breast cancer under chemotherapy OR under therapy with aromatase inhibitors or anti-oestrogens (currently and/or up to 3 months after the completion of the therapy) AND the symptoms of vulvovaginal dryness, which started with the beginning of the tumour therapy<br>- Written Declaration of Consent for the voluntary participation in the study is available

Synopsis (continued)

Exclusion criteria	- Women who suffered from the symptoms of vulvovaginal dryness prior to the beginning of the tumour therapy<br>- Patients undergoing radiation therapy<br>- Patients with other tumours<br>- Currently present vaginal infection<br>- Diseases of vulva or vagina<br>- Current additional therapy of vulvovaginal dryness or vulvovaginal atrophy<br>- Known hypersensitivity to one of the ingredients of Vagisan® Moisturising Cream<br>- Women not able to participate properly in this study<br>- Current alcohol and/or drug abuse<br>- Pregnant or lactating women
Medical device	Vagisan® Moisturising Cream (Dr. August Wolff Arzneimittel)
Posoloy and administration	Intravaginal:<br>1st Week: daily 1 x 2.5 g (half an applicator filling) in the evening<br>2nd to 4th week: daily or less frequently as needed, 1 x 2.5 g in the evening<br>On the external genital area:<br>Continuously for 4 weeks as needed, up to several times per day, about 0.5 g (1 fingertip unit) each time.
Total duration of the study	Approximately 6 months (recruiting phase: approximately five months)<br>Planned beginning: 04.01.2010<br>Planned ending: 30.06.2010
Duration of the application (for the patient)	4 weeks
Procedure	Day 0: Prior to the beginning of using Vagisan® Moisturising Cream<br>Visit 1:<br>1. Doctor<br>- Gynaecological examination<br>Recording of objective findings: Thinning of the vaginal epithelium, redness, petechial bleeding, discharge; assessment of the degree of severity using a scale of 0 to 4 (0 = none, 1 = slightly pronounced, 2 = moderately pronounced, 3 = very pronounced, 4 = severely pronounced)<br>- Medical documentation of the malignant disease by the doctor (diagnosis) and, if applicable, of further diseases; documentation of the oncological therapy and, if applicable, of other medication (current)

Synopsis (continued)

Procedure (continued)	2. Patient questionnaire<br>- Recording of subjective symptoms: feeling of dryness, itching, burning, pain independent on sexual intercourse, pain during sexual intercourse; assessment of the degree of severity using a scale of 0 to 4 (0 = none, 1 = slightly pronounced, 2 = moderately pronounced, 3 = very pronounced, 4 = severely pronounced), urinary incontinence<br>- Indication of the time these symptoms started<br>- Indication whether any medications against vaginal dryness were used before. If yes, name of the medication. General evaluation of the efficacy and tolerability, each with the "grades" 1-6 (1 = very good to 6 = unsatisfactory)<br><br>Day 0 to Day 28: Application of Vagisan® Moisturising Cream<br><br>Day 28: Study completion<br>Visit 2<br>1. Doctor<br>- Final gynaecological examination with assessmentof findings (similar to Day 0)<br>- Medical compilation of the history of any other diseases and medications taken within the last 4 weeks<br>- Medical documentation and assessment by the doctor<br>o Recording of known side effects (predefined parameters): "Temporary itching or burning (under 10 minutes)", "longer lasting itching or burning (10 to 60 minutes)", "strong intolerability (itching, burning, swelling longer than 60 minutes)", "discharge of cream".<br>o Questioning patients about SAEs and AEs, assessment of the causal relation by the doctor and, in the case of positive causality (see Section 8), documentation as a side effect of the product (adverse medical device effect, ADME).<br>- Medical evaluation<br>o General assessment of tolerability ("grades" 1-6)*<br>o General assessment of efficacy ("grades" 1-6)*<br>o Comprehensive Evaluation of Vagisan® Moisturising Cream Medical Device ("Grades" 1-6)*<br>* 1 = very good to 6 = unsatisfactory

Synopsis (continued)

Procedure (continued)	2. Patient questionnaire<br>- Frequency of intravaginal application during the 2nd week to the 4th week<br>- Frequency of application to the external genital area<br>- Recording of subjective symptoms: feeling of dryness, itching, burning, pain independent on sexual intercourse, pain during sexual intercourse; assessment of the degree of severity using a scale of 0 to 4 (0 = none, 1 = slightly pronounced, 2 = moderately pronounced, 3 = very pronounced, 4 = severely pronounced), urinary incontinence<br>- General assessment of tolerability ("grades" 1-6)*<br>- General assessment of efficacy ("grades" 1-6)*<br>- Comprehensive Evaluation of the Vagisan® Moisturising Cream Medical Device("Grades" 1-6)*<br>* 1 = very good to 6 = unsatisfactory
Statistics	Only descriptive statistics<br><br>Evaluation of the following parameters:<br>- Type and frequency of SAEs<br>- Type and frequency of side effects (adverse medical device events)<br>- General assessments of tolerability (doctor / patient)<br>- Subjective symptom score (individual scores, sum of scores; for each symptom, change day 0 minus day 28)<br>- Objective findings score (individual scores, sum of scores; for each finding, change day 0 minus day 28)<br>- General assessments of efficacy (doctor / patient)<br>- Comprehensive evaluation of the Vagisan® Moisturising Cream medical device (doctor / patient)<br>- Medical history data of the patient on vulvovaginal dryness (duration, previous treatment) and incontinence<br>- Frequency of usage of Vagisan® Moisturising Cream, intravaginally and on external genital area<br>- Tumour illness (diagnosis) and, if applicable, further illnesses (in the medical history and during the study)<br>- Type of tumour therapy and, if applicable, further medications (in the medical history and during the study)

3. Introduction

Hormone-related changes play an important role in the ageing process of the skin. If oestrogens are lacking, signs of atrophy are also visible on the vaginal skin. An oestrogen deficit occurs physiologically during post-menopause, but also occasionally occurs in pre-menopausal women. In particular, symptoms of an oestrogen deficit may also be noticed on the vaginal skin after the ovariectomy surgery (removal of the ovaries). The collagen and water content in the vaginal skin decreases. It is less elastic and more susceptible to infections. The epithelialium becomes thinner, red and susceptible to injuries; bleedings can occur (petechial bleeding). Occasionally, there is a discharge as a reaction to this condition.

Subjectively, the atrophy of the vaginal epithelium very frequently leads to the symptom of dryness, itching, burning and pain in the area of the vagina and/or vulva - and also results in dyspareunia (pain during sexual intercourse). These symptoms may significantly impair patient's quality of life.

Also independent of the hormone status, the subjective symptoms of the vulvovaginal dryness may occur, for example, as a side effect of systemically applied pharmaceuticals of different substance classes. These symptoms are frequently observed during chemotherapy, as well as under tumour treatment with aromatase inhibitors or anti-oestrogens. Changes to the vaginal skin, such as redness and petechial bleeding, may be also determined objectively. Moreover, subjective symptoms of a dry and sensitive vagina and vulva may also exist without any objectified background.

Normally, vaginal oestrogen therapy is executed with good treatment success in post-menopausal women or in women after ovariectomy surgery. However, there are situations when oestrogen therapy is contra-indicated or not desired.

In order to improve the moisture balance in the vagina, the regeneration of the vaginal mucous membranes or to reduce the vaginal symptoms, different hormone-free medical devices are available. These are normally gel-based products. Prior to the introduction of the hormone-free Vagisan® Moisturising Cream medical deviceinto the (German) market, there was no hormone-free cream for intravaginal use for the treatment of vaginal drynessin patients with the described symptoms.

In a placebo-controlled, double blind study with Linoladiol® N Cream (estradiol-containing cream for the treatment of vulvovaginal atrophy; Dr. August Wolff Arzneimittel), there was no statistically significant difference determined between verum and the placebo (active ingredient-free cream base) with regard to the improvement of subjective symptoms. This was the starting point for the development of Vagisan® Moisturising Cream, which is only different from the Linoladiol N-cream base through the addition of a lactic acid buffer and the content of benzyl alcohol (2% instead of previously 1%).

The addition of the lactic acid buffer causes a pH value of 4.5 in the cream and has the objective of maintaining or restoring a physiological pH value in the vaginal environment (which is at pH 4.0 - pH 4.4 for healthy pre-menopausal women).

Vagisan® Moisturising Cream (Dr. August Wolff Arzneimittel) is a certified class II b medical device available in the (German) market. The usage of Vagisan® Moisturising Cream is a purely symptomatic therapy where the skin of the vagina and on the vulva is provided with moisture and soothing lipids (cream).

Existing studies

Pre-clinical studies (pharmacological, pharmacokinetic and toxicological studies) are not available for the active ingredient-free Vagisan® Moisturising Cream medical device. However, all of the substances used in Vagisan® Moisturising Cream are already used as components in a variety of approved vaginal creams (pharmaceuticals / medicinal products) and therefore have already been investigated multiple times in preclinical and clinical studies.

A prospective, multicentric, controlled, randomized study to examine the efficacy and tolerability of Vagisan® Moisturising Cream (test medical device) in comparison with Gynomunal® vaginal gel (reference medical device) in a 2-period changeover design on n=120 patients with vaginal dryness who cannot or do not wish to use oestrogens, was executed in 2008. An inspection and critical evaluation of the relevant scientific literature was executed before that study.

The superiority of Vagisan® Moisturising Cream compared to Gynomunal® Vaginal Gel was proven for the primary endpoint "Improvement of subjective symptoms" in a statistically guaranteed manner (5% level). In addition to an improvement of the subjective symptoms, also an improvement of the objective findings of vaginal dryness was established.

After the usage of Vagisan® Moisturising Cream or Gynomunal® Vaginal Gel, there was frequently (with a calculated probability of 0.04 or 0.08) a temporary burning feeling or itching; for one patient in each treatment group this reaction lasted longer than 12 hours, and was classified as "intolerability". In the overall assessment on the tolerability of the products, the patients and the treating doctors evaluated Vagisan® Moisturising Cream significantly better than Gynomunal® Vaginal Gel ($p < 0.01$).

Product information

Ingredients
Benzyl alcohol, cetyl palmitate, cetyl stearyl alcohol, lactic acid, sodium lactate, octyldodecanol, polysorbate 60, sorbitan stearate, purified water. Vagisan Moisturising Cream contains no fragrances.

Vagisan® Moisturising Cream is a smooth, white cream that was developed to be used for the symptoms of vaginal dryness (vulvovaginal dryness). Vagisan® Moisturising Cream contains no hormones (oestrogens) and can therefore be used alongside hormone replacement therapy or be alternated with creams or vaginal pessaries that do contain hormones (oestrogens).

Due to the addition of lactic acid, the cream has a pH of 4.5. As a result, Vagisan® Moisturising Cream helps maintain a natural pH within the vagina. Two effects contribute to the relief of symptoms of vaginal dryness with Vagisan® Moisturising Cream: The cream has a high water content, thereby moisturising the skin of the vaginal and external genital area; soothing lipids keep the skin smooth.

Regular use of Vagisan® Moisturising Cream can help prevent irritation and inflammation within the vagina and external genital area. The cream can also be used for vaginal dryness before sexual intercourse, including with latex condoms. For women wishing to conceive, there are no contraindications to using Vagisan® Moisturising Cream for vaginal dryness, since sperm motility is not impaired. The cream may also be applied during menstrual periods.

Application of Vagisan Moisturising Cream

Vagisan Moisturising Cream is intended for application in the vagina and to the external genital area. Vagisan Moisturising Cream can be applied with or without an applicator.

For symptoms in the vaginal entrance and external genital area, use a clean finger to apply and spread the cream as needed; a ribbon of cream about 0.5 cm long is sufficient (1 fingertip unit). To make it easier to insert the applicator, a small amount of cream can also be applied to the area around the vaginal entrance.

For vaginal symptoms, Vagisan® Moisturising Cream is inserted into the vagina with the help of the included applicator; in the beginning every evening before going to bed. To apply, fill the applicator halfway (about 2.5 g cream). Once symptoms have improved, the application can be reduced as needed.

Possible side effects

Occasionally, due to the lactic acid additive, temporary mild local irritation (itching, stinging) and a slight discharge may occur in some cases. Hypersensitivity reactions (for example, redness, stinging) may occur very rarely.

Restrictions for use

Vagisan Moisturising Cream must not be used in known hypersensitivity or allergy to any of the ingredients. Do not use Vagisan Moisturising Cream if you have a vaginal infection.

Risk/benefit assessment

All of the substances used in Vagisan® Moisturising Cream are known and are used in a number of approved medicinal products. A clinical study with Vagisan® Moisturising Cream with 92 women with symptoms of vulvovaginal dryness showed good efficacy (in terms of a relief of subjective symptoms and improvement of objective findings) and tolerability.

There may be a temporary mild feeling of stinging or itching after application due to the lactic acid additive. As with any product applied to the skin and mucous membranes, it cannot be ruled out - in terms of a potential risk – that local intolerability (including allergic reaction) may occur in individual cases.

A risk analysis according to the directives applicable to medical devices was executed. No relevant hazards were identified on the basis of the risk analysis for the Vagisan® Moisturising Cream medical device. Vagisan® Moisturising Cream can be evaluated as an effective and safe medical device. The benefits of the product for the user clearly exceed the potential risk.

4. Objective of the application study

The objective of this application study is to investigate whether the use of Vagisan® Moisturising Cream in breast cancer patients undergoing chemotherapy or therapy with anti-oestrogens / aromatase inhibitors may induce an improvement of the subjective symptoms and objective findings of vulvovaginal dryness, while also offering a good tolerability.

Vagisan® Moisturising Cream should be examined for the following parameters:

Tolerability

- Type and frequency of side effects (adverse medical device effects, AMDE)
- General assessments of tolerability (doctor / patient)

Efficacy

- Subjective symptoms (feeling of dryness; itching; stinging; pain independent of sexual intercourse; pain during sexual intercourse; urinary incontinence)
- Objective findings (thinning of the vaginal epithelium; redness; petechial bleeding; discharge)
- General assessments of efficacy (doctor / patient)

Overall assessment of the medical device

- Comprehensive evaluation of Vagisan® Moisturising Cream medical device (doctor / patient)

5. Quality assurance

This application study considers, as far as possible, the "recommendations for the execution of application observations" published in 1997 by the "German Association for Medical Information Systems, Biometry and Epidemiology" (GMDS) as well as the published draft of "Mutual recommendations for planning, execution and analysis of application observations" by the German Federal Institute for Drugs and Medical Devices (BfArM) and the Paul Ehrlich Institute (PEI) on May 09, 2007.

The following preliminary special measures have to provide for the quality assurance:

- Description and communication of the special importance of each person participating in the application study to reach high quality data within the scope of an application study.

- Exact procedural rules for all steps of the application study, in particular with regard to the aspects of pharmacovigilance.

The documentation on the patient data is carried out online through electronic documentation surveys (eCRFs = electronic case report forms). The doctor must ensure complete documentation. Electronically recorded investigation data are automatically examined by the RDE system for completeness and plausibility.

Immediately after the detection of incomplete or implausible information that affects the medical device safety will directly contact the doctor. Medical device safety relevant events are documented on the basis of SAEs or evaluated adverse events (definition of SAE or AE see Section 8).

6. Observation scope / Organization

In the period from 04.01.2010 to 30.06.2010, 150 pre-menopausal patients above the age of 18 with breast cancer under chemotherapy OR therapy with anti-oestrogens or aromatase inhibitors (current therapy or up to 3 months after completion of the therapy) AND with the symptoms of vulvovaginal dryness, should be included in the application study. The patient number of 150 was seen as sufficient for determining validly whether an improvement of the subjective symptoms and the objective findings of vulvovaginal dryness can be achieved with good tolerability. Each patient will be treated for 28 days with Vagisan® Moisturising Cream.

Women, who suffered from the symptoms of vulvovaginal dryness prior to the beginning of tumour therapy, should not participate in the application study. Patients with tumours, other than those of breast cancer and patients, who are receiving a radiation therapy, are excluded from the application study. Illnesses of the vagina or vulva, as well as current additional therapies for vulvovaginal dryness or vulvovaginal atrophy, are also criteria for the exclusion. Patients with proven hypersensitivity to one of the ingredients of Vagisan® Moisturising Cream or current existing vaginal infection may not participate in the application study. Women, incapable to participate properly in the study according to the requirements of the study or currently suffering from alcohol or drug abuse, may not participate in the application study. Pregnant or breastfeeding women may not participate in this application study.

This multicentric application study will be carried out in gynaecology clinics.

Vagisan® Moisturising Cream medical device and the applicators required for the usage are provided by the sponsor, Dr. August Wolff Arzneimittel.

Prior to the participation of in the application study, the Declaration with the clarification of the investigator’s (doctor's) participation must be completed and signed. . Before a patient can be included in the study, the investigator (doctor) must have a signed patient's consent to participate in the application study after a relevant explanation by the doctor.

The documents for the application study are provided to each participating doctor by . The documentation of the patient data is carried out online through electronic health records surveys (eCRFs = electronic case report forms). After signing of the participation declaration, the participating doctor will receive an access code from , which provides for the electronic access to the documentation. The online documentation should be processed as soon as possible after the patient has been included in the application study.

Any extra work required by the doctors for the completion of the electronic documentation surveys within the scope of this application study will be paid on the basis of the German scale of medical fees (GOÄ). Fully completed case report forms shall be compensated with 120 € (net) per patient.

This application study will neither impact the treatment recommendations, doctor's instructions or restrict the therapy freedom. The treatment must be carried out in agreement with the instructions of use for Vagisan® Moisturising Cream and should correspond to the individual medical condition.

7. Observation procedure

The instructions for use of Vagisan® Moisturising Cream are explicitly referred to. These instructions contain all important information (such as application areas, side effects, interactions, contraindications, type and duration of usage, dosage).

Prior to the start of the study, the patient will receive oral and written information from the participating doctor about the procedure of the application study. All potential study participants must provide their written consent to the voluntary participation in this application study before they may be included.

The suitability of the patient for the participation in this application study will be examined based on the inclusion and exclusion criteria.

Upon inclusion in the application study, each patient will be assigned an individual patient number, used to identify the documents of the patient. All participating doctors are obligated to make a note in the patient documents as to the patient's participation in this application study. Medical information about patients should only be forwarded and analysed in an anonymous form. The identity of all patients participating in this application study must be treated confidentially. In the event of inquiries, particularly in connection to the reports on side effects, the doctor must be able to remove the anonymization of the patient observed in this application study. The patient number and name are entered in the patient identification list for this purpose. This remains in the office and must also be treated confidentially.

Visit 1: Day 0, prior to usage of Vagisan® Moisturising Cream

1. Doctor
   - Gynaecological examination

     Recording of objective findings: Thinning of the vaginal epithelium, redness, petechial bleeding, discharge; assessment of the degree of severity using a scale of 0 to 4 (0 = none, 1 = slightly pronounced, 2 = moderately pronounced, 3 = very pronounced, 4 = severely pronounced)
   - Medical documentation of the malignant disease by the doctor (breast cancer) and, if applicable, of further diseases. Documentation of the oncological therapy and, if applicable, of other non-oncological medication (current)

2. Patient questionnaire
   - Recording of subjective symptoms: feeling of dryness, itching, burning, pain independent on sexual intercourse, pain during sexual intercourse; assessment of the degree of severity using a scale of 0 to 4 (0 = none, 1 = slightly pronounced, 2 = moderately pronounced, 3 = very pronounced, 4 = severely pronounced), urinary incontinence
   - In each case with the specification of the start date of the symptoms

- Indication whether any medications against vaginal dryness were used before. If yes, name of the medication. General evaluation of the efficacy and tolerability (in each case with the "grades" 1-6) (1 = very good to 6 = unsatisfactory)

Day 0 - 28: Application of Vagisan® Moisturising Cream

Intravaginally:

1st week: daily 1 x 2.5 g (half an applicator filling) in the evening

2nd to 4th week: daily or less as needed, 1 x 2.5 g in the evening

On the external genital area:

Continuously for 4 weeks as needed, also several times per day, about 0.5 g (1 fingertip unit) each time.

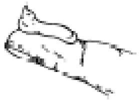

Visit 2: Day 28, completion of the study

1. Doctor
   - Final gynaecological examination with assessment of findings (similar to day 0)
   - Doctor compiles the information about any other diseases and medications taken within the last 4 weeks
   - Recording of known adverse side effects due to the application of Vagisan® Moisturising Cream medical device (by patient interview):
     - Predefined parameters: "Temporary itching or stinging (under 10 minutes)", longer lasting itching or stinging (10 to 60 minutes)", "strong intolerability (itching, stinging, swelling longer than 60 minutes", "discharge of cream"
     - Questioning patient about SAEs and AEs; doctor's assessment of the causal relationship and, in the case of positive causality (see Section 8), documentation as side effect (adverse medical device effect).
   - Assessment performed by the doctor:
     - General assessment of tolerability ("grades" 1-6)*
     - General assessment of efficacy ("grades" 1-6)*
     - Comprehensive Evaluation of the Vagisan® Moisturising Cream Medical Device ("Grades" 1-6)*

       * 1 = very good to 6 = unsatisfactory

2. Patient questionnaire
   - Frequency of intravaginal application during the 2nd week to the 4th week
   - Frequency of application to the external genital area
   - Recording of subjective symptoms: feeling of dryness, itching, burning, pain independent on sexual intercourse, pain during sexual intercourse; assessment of the degree of severity using a scale of 0 to 4 (0 = none, 1 = slightly pronounced, 2 = moderately pronounced, 3 = very pronounced, 4 = severely pronounced), incontinence
   - General assessment of tolerability ("grades" 1-6)*
   - General assessment of efficacy ("grades" 1-6)*
   - Comprehensive Evaluation of Vagisan® Moisturising Cream Medical Device ("Grades" 1-6)*

   * 1 = very good to 6 = unsatisfactory

8. Definitions for medical device safety

Adverse events[1]

An adverse event is any damaging event that a patient experiences after administration of a medical device independent of whether a causal connection with this treatment is suspected or if proper usage was exercised.

Adverse medical device effect[1] (AMDE)

An adverse medical device effect is to be understood here as a synonym for the terms "side effect" and "suspicion of an adverse medical device effect". Side effects are harmful, unintentional reactions that occur with the proper usage of a medical device.

An adverse medical device effect, in contrast to an adverse event, is characterized through the fact that there is the suspicion of a causal connection between the administered medical device and the observed event; this means the connection must at least be classified as "unlikely" by the treating doctor.

Serious side effects / serious adverse events[1] / incidents

Serious side effects / adverse events (SAEs; English: serious adverse events = SAEs) are events that are fatal or life-threatening, require hospitalisation or extension of hospital treatment, lead to permanent or severe impairment, disability, congenital anomalies or birth defects, or events that are medically important.

Serious adverse events are a synonym for the term "incidents" (in accordance with the medical device safety regulation) and include an adverse medical device event that led, could have led or could lead directly or indirectly to death or serious worsening of the state of health of a patient, user or another person.

Medically important side effects / adverse events are events that are not immediately fatal or life-threatening or lead to hospitalisation, but significantly impair the patient or require an intervention / treatment in order to prevent a condition that corresponds with the definition for "serious" (for example, events that require measures to prevent hospitalisation).

---

[1] - Definitions in accordance with Section 4 (13) AMG (German Pharmaceuticals Act) and “Fifth announcement on the notification of side effects and pharmaceutical abuse according to Section 63b, Para 1 - 8 of the German Pharmaceuticals Act” of 05.12.2007.

In cases of known or suspected teratogenic effect of the preparation also pregnancy is a serious adverse event.
An unplanned pregnancy always fulfils the criteria of a serious adverse event.

Normally, hospitalisation is defined as an inpatient treatment which includes an overnight stay. Inpatient (hospital) treatment for purely diagnostic reasons, routine control examinations or surgical interventions already planned before the beginning of the application study, as well as inpatient treatmens due to social indications do not qualify the hospitalisation in the sense of the term "serious".

Evaluation of the causal connection

(According to WHO-UMC Causality Categories [UMC = Uppsala Monitoring Centre])

Causal connection "certain" (UMC – certain)
A clinical event, including abnormal laboratory findings, that occur within a plausible time frame associated with the administration of a medical device and cannot be explained through a concomitant disease or other medications or chemicals. The reaction after discontinued usage of the medical device (dechallenge) should be clinically plausible. The event must be clear in a pharmacological or phenomenological sense; a suitable re-exposition procedure (rechallenge) should be used to show this.

Causal connection "probable" (UMC – probable/likely)
A clinical event, including abnormal laboratory findings, that occur in the relevant time frame associated with the administration of a medical device and probably cannot be explained through a concomitant disease or other medications or chemicals, whereby the discontinued usage of the medical device (dechallenge) causes a clinically plausible reaction. Specifications about a re-exposition are not required in order to comply with this definition.

Causal connection "possible" (UMC – possible)
A clinical event, including abnormal laboratory findings, that occur in the relevant time frame associated with the administration of a medical device and could also be explained through a concomitant disease or other medications or chemicals. Information about the reaction after discontinuing the usage of the medical device may be missing or unclear.

Causal connection "unlikely" (UMC – unlikely)
A clinical event, including abnormal laboratory findings, that coincide with the administration of a medical device (temporal connection), whereby a causal connection is unlikely due to the time frame and other medications, chemicals or basic illnesses represent plausible explanations.

Causal connection "no connection"
All unintended reactions with evidently different, internal or external causes, other than the administration of the medical device, are ruled out as a suspicion for a side effect. This includes symptoms that are a clear expression of the patient's basic illness or a concomitant disease. If the symptoms worsen, the possibility of a similarity between the illness symptoms and side effects should be examined closely in order to see if a side effect exists due to the medical device and, when in doubt, a report should be filed.

Symptoms that demonstrably occurred in the same (or stronger) intensity before the administration of the medical device or cases in which the named medical device was proven to not have been used, should be ruled out as cases of suspicion for side effects.

Causal connection "unclassified" (UMC - conditional/unclassified)
A clinical event, including abnormal laboratory findings, that was reported as an adverse medical device effect, for which additional data are required for an appropriate evaluation or for which additional data are currently being examined.

The following definition is additionally included under "unclassifiable" (UMC - unassessible/unclassifiable):
A report that suggests an adverse medical device effect that cannot be evaluated, because the available information is insufficient or contradictory and cannot be amended or verified.

9. Report or documentation of SAE, adverse medical device effects (AMDE), including suspected adverse medical device effects

Since this application study comprises a documentation in terms of the normal application area of the certified Vagisan® Moisturising Cream medical device, only "serious adverse events" (serious side effects / serious adverse events[1] / incidents; for definition see Chapter 8), adverse medical device effects, as well as cases of suspicion of medical device side effects are documented. Thus, the causal connection of an "adverse event" with the medication must be evaluated by the doctor at least as "unlikely" (exact definitions see Chapter 8) in order to make an "adverse event" into an “AMDE” (adverse medical device effect). Explicitely, events that are not serious and that do not have any recognizable causal connection with the medication are not listed separately.

Adverse medical device effects, as well as cases of suspicion of adverse medical device effects (Causality from the doctor at least evaluated as "unlikely"), are recorded and documented within the scope of the 2nd visit upon completion of the study.

Serious adverse events (serious side effects / serious adverse events[1] / incidents; definition see Chapter 8), which occur within the scope of the application study, must be immediately documented upon detection and forwarded by the doctor to within 24 hours:

For the documentation the following forms are provided: "Reporting serious adverse events (SAEs)" and "Report on suspected cases of adverse medical device effects".

If a SAE occurs during the application study that requires early termination of the application study, this will be documented by the doctor and forwarded to within 24 hours upon detection. must forward SAEs to the sponsor immediately upon detection.

10. Data management and biometric analysis

The documentation of the findings during the study is supported by computer through an Online Remote Data Entry (RDE) application, which is developed by according to the case report form (CRF) generated by . The access rights to this online data entry system are controlled through access codes. Entries and corrections may only be executed by the participating doctors and their authorised employees. Corrections are made so that the old entry can still be retrieved. All entries and corrections are automatically recorded with the date, time and name of the person making the entry ("audit trail").

The computer supported data entry by the doctor is carried out with plausibility checks, corresponds to the requirements of the FDA and the EMEA and fulfils the highest quality standards.

The participating doctor must keep a patient identification list for the purpose of patient identification. The participation in the application study (patient number/beginning and end of the participation/medical device) must also be listed in the doctor's patient files.

A descriptive statistic is used in this application investigation.

Evaluation of the following parameters:

- Type and frequency of SAEs
- Type and frequency of side effects (adverse medical device effects as well as cases of suspicion of adverse medical device effects)
- General assessments of tolerability (doctor / patient)

- Subjective symptom score (individual scores, sum of scores; each change day 0 minus day 28)
- Objective findings score (individual scores, sum of scores; for each symptom change day 0 minus day 28)
- General assessments of efficacy (doctor / patient)
- Comprehensive evaluation of Vagisan® Moisturising Cream medical device (doctor / patient)

- Medical history data of the patient on vulvovaginal dryness (duration, previous treatment), urinary incontinence
- Frequency of usage of Vagisan® Moisturising Cream, intravaginally and on external genital area
- Tumour illness (diagnosis) and, if applicable, further illnesses (in the medical history and during the study)
- Type of tumour therapy and, if applicable, further medications (in the medical history and during the study)

11. Duty of notification

Before the beginning of the application study, the approval of the International Freiburg Ethics Committee must be obtained.

Since this multicentric application study is not a clinical study in accordance with MPG (Medical Devices Act) and in accordance with 93-42-EWG directive, there is no obligation of notifying the competent authority. Nevertheless, the regional authorities responsible for clinical studies with medical devices will be informed about the execution of this application study. The notification includes the announcement of the application study, as well as the indication of the names of the participating doctors. Since Vagisan® Moisturising Cream is a non-prescription and non-refundable medical device and the required preparations are provided to the participants of the investigation by the sponsor, the application study will not be reported to the The National Association of Statutory Health Insurance Physicians and the Federal Associations of Health Insurance Funds.

After completion of the application study, a detailed report will be created, which includes a statistical analysis, as well as an evaluation of the results from a medical point of view. The accuracy of the content will be confirmed by the signatures of the scientific supervisor of the application study, the sponsor's project manager, the biometrician responsible for statistical analysis and the employees of responsible for reporting.

12. Bibliography

1. Mazur D; Vens-Cappell B; Lohmann K; Breckwoldt M: Fraktionierte Anwendung einer 17beta Estradiolcreme zur Behandlung der atrophischen Kolpitis postmenopausaler Frauen. *Geburtshilfe und Frauenheilkunde; 65/6, 584-589 (2005)*

2. SOGC Clinical Practice Guidelines: The detection and management of vaginal atrophy International. *Journal of Gynecology and Obstetrics; 88/2, 222-228 (2005)*

3. Bachmann GA, Nevadunsky NS: Diagnosis and Treatment of Atrophic Vaginitis. *American Family Physician. 2000 May 15;61(10):3090-6*

4. Mok K, Juraskova I, Friedlander M: The impact of aromatase inhibitors on sexual functioning: Current knowledge and future research directions. *The Breast;17, 436- 440 (2008)*

5. Bruno D, Feeney KJ: Management of postmenopausal symptoms in breast cancer survivors. Semin Oncol; 33:696-707 (2006)

6. Dr. August Wolff GmbH & Co. KG Arzneimittel: Vagisan® Moisturising Cream – Package Leaflet *Revision date: March 2009*

7. Dr. August Wolff GmbH & Co. KG Arzneimittel: Vagisan® Moisturising Cream - Study on Efficacy and Tolerability. *Synopsis of the study report, Dr. August Wolff (2008)*

13. Signatures

Scientific Supervisor of the Application Study: ______________________ ____________
Date

Sponsor's Project Manager: ______________________ ____________
Date

Sponsor’s Medical Advisor Gynaecology: ______________________ ____________
Date

Head of Monitoring at : ______________________ ____________
Date

Biometrician and project director at : ______________________ ____________
Date
