# Supplementary material for: Topical treatment of vaginal dryness with a non-hormonal cream in women undergoing breast cancer treatment - An open prospective multicenter study
Source: PLoS One. 2019 Jan 24;14(1):e0210967. doi: 10.1371/journal.pone.0210967 (PMC6345451; doi:10.1371/journal.pone.0210967)
Supplement: S3 File — (PDF) [file pone.0210967.s004.pdf]

Multizentrische Untersuchung zur Anwendung von Vagisan® FeuchtCreme bei Brustkrebs-Patientinnen mit dem Beschwerdebild der vulvovaginalen Trockenheit

BEOBACHTUNGSPLAN

Die in diesem Dokument enthaltenen Informationen sind **streng vertraulich** zu behandeln und dürfen nicht an Unbeteiligte weitergegeben werden.

Inhaltsverzeichnis

1. ANSCHRIFTEN UND VERANTWORTLICHKEITEN 1

2. SYNOPSIS 3

3. EINLEITUNG 7

4. ZIEL DER ANWENDUNGSUNTERSUCHUNG 11

5. QUALITÄTSSICHERUNG 12

6. BEOBACHTUNGSUMFANG/ORGANISATION 13

7. BEOBACHTUNGSABLAUF 15

8. DEFINITIONEN ZUR MEDIZINPRODUKTSICHERHEIT 18

9. MELDUNG BZW. DOKUMENTATION VON SUE, UNERWÜNSCHTEN MEDIZINPRODUKTWIRKUNGEN (UMW) SOWIE VERDACHTSFÄLLEN VON UMW 21

10. DATA MANAGEMENT UND BIOMETRISCHE AUSWERTUNG 22

11. ANZEIGEPFLICHT 24

12. LITERATUR 25

13. UNTERSCHRIFTEN 26

1. Anschriften und Verantwortlichkeiten

**Wissenschaftliche Leitung**	
**Sicherheitsbeauftragte für Medizinprodukte**	
**Medizinische Expertin Gynäkologie beim Sponsor**	
**Biometrie und Projektleiter ,**<br>**Berichterstellung**	
**Monitoring**	

Anschriften und Verantwortlichkeiten (Fortsetzung)

**Sponsor**	
**Projektleiter beim Sponsor**	

2. Synopsis

Titel der Studie	Multizentrische Untersuchung zur Anwendung von Vagisan® FeuchtCreme bei Brustkrebs-Patientinnen mit dem Beschwerdebild der vulvovaginalen Trockenheit
Studienziel	Untersuchung der Anwendbarkeit von Vagisan® FeuchtCreme bei Brustkrebs-Patientinnen mit der Zielfrage, ob eine Besserung der subjektiven Beschwerden und des objektiven Befundes einer vulvovaginalen Trockenheit bei guter Verträglichkeit erreicht werden kann
Zielgruppe	Brustkrebs-Patientinnen mit dem Beschwerdebild der vulvovaginalen Trockenheit (häufige Begleiterscheinungen bei oder nach onkologisch-therapeutischen Maßnahmen wie Chemotherapie oder Einnahme von anderen Arzneimittel wie z.B. Aromatasehemmer, Antiestrogene)
Untersuchungsparameter	Verträglichkeit<br>- Art und Häufigkeit von Nebenwirkungen (Unerwünschte Medizinproduktwirkungen, UMW)<br>- Allgemeines Urteil zur Verträglichkeit (Arzt / Patientin)<br>Wirksamkeit<br>- Subjektive Beschwerden (Trockenheitsgefühl; Juckreiz; Brennen; Schmerzen unabhängig vom Geschlechtsverkehr; Schmerzen beim Geschlechtsverkehr, Inkontinenz)<br>- Objektive Befunde (Verdünnung des Vaginalepithels; Rötung; petechiale Blutungen; Fluor)<br>- Allgemeines Urteil zur Wirksamkeit (Arzt / Patientin)<br>Gesamtbewertung des Medizinproduktes<br>- Zusammenfassende Beurteilung des Medizinproduktes Vagisan® FeuchtCreme (Arzt / Patientin)
Studiendesign	Multizentrische Anwendungsuntersuchung mit einem Medizinprodukt
Zentren	Gynäkologische Praxen
Zahl der Zentren	Ca. 30 Praxen mit jeweils mindestens 5 Patientinnen
Zahl der Patientinnen	N = 150
Einschlusskriterien	- Frauen ab 18 Jahre<br>- Prämenopausale Frauen mit Mamma-Carcinom unter Chemotherapie ODER unter Therapie mit Aromatasehemmern oder Antiöstrogenen (jeweils aktuell bzw. bis zu 3 Monate nach Beendigung der Therapie) UND dem seit Beginn der Tumortherapie einsetzenden Beschwerdebild der vulvovaginalen Trockenheit<br>- Schriftliche Einverständniserklärung zur freiwilligen Teilnahme an der Studie liegt vor

Synopsis (Fortsetzung)

Ausschlusskriterien	- Frauen, die bereits vor dem Beginn der Tumorbehandlung das Beschwerdebild der vulvovaginalen Trockenheit hatten<br>- Patientinnen, die eine Bestrahlungstherapie erhalten<br>- Patientinnen mit weiteren Tumoren<br>- Aktuell vorliegende Vaginalinfektion<br>- Erkrankungen an der Vulva oder Vagina<br>- Aktuelle zusätzliche Therapie der vulvovaginalen Trockenheit oder vulvovaginalen Atrophie<br>- Bekannte Überempfindlichkeit gegenüber einem der Inhaltsstoffe von Vagisan® FeuchtCreme<br>- Frauen, die nicht in der Lage sind die Studie ordnungsgemäß durchzuführen<br>- Aktueller Alkohol- und oder Drogenabusus<br>- Frauen in der Schwangerschaft oder Stillzeit
Medizinprodukt	Vagisan® FeuchtCreme (Dr. August Wolff Arzneimittel)
Dosierung und Anwendung	Intravaginal:<br>1. Woche: täglich 1 x 2.5 g (eine halbe Applikatorfüllung) abends<br>2. – 4. Woche: täglich oder nach Bedarf seltener, 1 x 2.5 g abends<br>Im äußeren Intimbereich:<br>durchgehend 4 Wochen nach Bedarf, auch mehrfach täglich, jeweils ca. 0.5 g (1 Fingertip-unit).
Gesamtdauer der Studie	Ca. 6 Monate (Rekrutierungsphase: ca. 5 Monate)<br>Geplanter Beginn: 04.01.2010<br>Geplantes Ende: 30.06.2010
Dauer der Anwendung (für die Patientin)	4 Wochen
Ablaufplan	<u>Tag 0: vor Beginn der Anwendung von Vagisan® FeuchtCreme</u><br><u>Visite 1:</u><br>1. Arzt<br>- Gynäkologische Untersuchung<br>Erhebung der objektiven Befunde: Verdünnung des Vaginal-epithels, Rötung, petechiale Blutungen, Fluor; Bewertung des Schweregrades anhand einer Skala von 0 bis 4 (0 = keine, 1 = gering, 2 = mäßig, 3 = stark, 4 = sehr stark ausgeprägt)<br>- Ärztliche Erfassung der malignen Erkrankung (Diagnose) und ggf. weiterer Erkrankungen, der onkologischen Therapie und ggf. weiterer Medikationen (aktuell)

Synopsis (Fortsetzung)

<table>
<tr><td>Ablaufplan (Fortsetzung)</td><td>
2. Patientinnen-Fragebogen
- Erhebung der subjektiven Beschwerden: Trockenheitsgefühl, Juckreiz, Brennen, Schmerzen unabhängig vom Geschlechtsverkehr, Schmerzen beim Geschlechtsverkehr; Bewertung des Schweregrades anhand einer Skala von 0 bis 4 (0 = keine, 1 = gering, 2 = mäßig, 3 = stark, 4 = sehr stark ausgeprägt), Harninkontinenz
- Jeweils Angabe, seit wann diese Beschwerden bestehen
- Angabe, ob bereits Präparate gegen Scheidentrockenheit angewendet wurden. Falls ja, Nennung des Präparates. Pauschale Bewertung der Wirksamkeit und Verträglichkeit, jeweils mit den „Noten“ 1-6 (1 = sehr gut bis 6 = ungenügend)

<u>Tag 0 bis Tag 28: Anwendung von Vagisan® FeuchtCreme</u>

<u>Tag 28: Abschluss der Untersuchung</u>
<u>Visite 2</u>
1. Arzt
- Gynäkologische Abschlussuntersuchung mit Befundung (wie an Tag 0)
- Ärztliche Erfassung jeglicher weiterer Erkrankungen und Medikationen in den letzten 4 Wochen
- Ärztliche Dokumentation und Beurteilung
  o Erfassung von bekannten Nebenwirkungen (vorgegebene Parameter): „Vorübergehendes Jucken oder Brennen (unter 10 Minuten)“, „länger anhaltendes Jucken oder Brennen (10 bis 60 Minuten)“, „starke Unverträglichkeit (Jucken, Brennen, Schwellung länger als 60 Minuten)“, „Ausfluss der Creme“.
  o Befragung der Patientin nach SUEs und UEs, ärztliche Bewertung des Kausalzusammenhangs und im Fall positiver Kausalität (s. Abschnitt 8) Dokumentation als Nebenwirkung (Unerwünschte Medizinproduktwirkung, UMW)
- Ärztliche Beurteilung
  o Allgemeines Urteil zur Verträglichkeit („Noten“ 1-6)*
  o Allgemeines Urteil zur Wirksamkeit („Noten“ 1-6)*
  o Zusammenfassende Beurteilung des Medizinproduktes Vagisan® FeuchtCreme („Noten“ 1-6)*
    * 1 = sehr gut bis 6 = ungenügend
</td></tr>
</table>

Synopsis (Fortsetzung)

Ablaufplan (Fortsetzung)	2. Patientinnen-Fragebogen<br>- Häufigkeit der intravaginalen Anwendungen in der 2. bis 4. Woche<br>- Häufigkeit der Anwendung im äußeren Intimbereich<br>- Erhebung der subjektiven Beschwerden: Trockenheitsgefühl, Juckreiz, Brennen, Schmerzen unabhängig vom Geschlechtsverkehr, Schmerzen beim Geschlechtsverkehr; Bewertung des Schweregrades anhand einer Skala von 0 bis 4 (0 = keine, 1 = gering, 2 = mäßig, 3 = stark, 4 = sehr stark ausgeprägt), Harninkontinenz<br>- Allgemeines Urteil zur Verträglichkeit („Noten“ 1-6)*<br>- Allgemeines Urteil zur Wirksamkeit(„Noten“ 1-6)*<br>- Zusammenfassende Beurteilung des Medizinproduktes Vagisan® FeuchtCreme („Noten“ 1-6)*<br>* 1 = sehr gut bis 6 = ungenügend
Statistik	Nur deskriptive Statistik<br><br>Auswertung folgender Parameter:<br>- Art und Häufigkeit von SUEs<br>- Art und Häufigkeit von Nebenwirkungen (Unerwünschten Medizinproduktwirkungen, UMW)<br>- Allgemeine Urteile zur Verträglichkeit (Arzt / Patientin)<br><br>- Subjektiver Beschwerde-Score (Einzelscores, Summenscore; jeweils Änderung Tag 0 minus Tag 28)<br>- Objektiver Befund-Score (Einzelscores, Summenscore; jeweils Änderung Tag 0 minus Tag 28)<br>- Allgemeine Urteile zur Wirksamkeit (Arzt / Patientin)<br>- Zusammenfassende Beurteilung des Medizinproduktes Vagisan® FeuchtCreme (Arzt / Patientin)<br><br>- Anamnestische Angaben der Patientin zur vulvovaginalen Trockenheit (Dauer, Vorbehandlung) und Inkontinenz<br>- Häufigkeit der Anwendung von Vagisan® FeuchtCreme, intravaginal und im äußeren Intimbereich<br>- Tumorerkrankung (Diagnose) und ggf. weitere Erkrankungen (anamnestisch und im Verlauf der Anwendungsuntersuchung)<br>- Art der Tumortherapie und ggf. weiterer Medikationen (anamnestisch und im Verlauf der Anwendungsuntersuchung)

3. Einleitung

Im Alterungsprozess der Haut spielen hormonell bedingte Veränderungen eine wichtige Rolle. Fehlen Östrogene, so werden Atrophie-Anzeichen auch an der Scheidenhaut sichtbar. Östrogenmangel tritt physiologischerweise in der Postmenopause auf, kommt aber auch gelegentlich bei prämenopausalen Frauen bereits vor. Insbesondere postoperativ nach Ovarektomie (Entfernung der Eierstöcke) können Östrogen-mangelerscheinungen sich auch an der Scheidenhaut bemerkbar machen. Der Kollagen- und Wassergehalt in der Scheidenhaut nimmt ab. Sie ist weniger elastisch und anfälliger für Infektionen. Das Epithel wird dünner, ist gerötet und leicht verletzlich; es kann punktuell zu Einblutungen kommen (petechiale Blutungen). Gelegentlich entsteht als Reaktion auf diesen Reizzustand auch Ausfluss (Fluor).

Subjektiv führt die Atrophie des Scheidenepithels sehr häufig zu Beschwerden von Trockenheit, Juckreiz, Brennen und Schmerzen im Bereich von Vagina und/oder Vulva – und in der Folge auch zu Dyspareunie (Schmerzen beim Geschlechtsverkehr). Insgesamt kann durch diese Symptome die Lebensqualität erheblich beeinträchtigt werden.

Auch unabhängig vom Hormonstatus kann das subjektive Beschwerdebild der vulvovaginalen Trockenheit auftreten, z.B. als Nebenwirkung von systemisch angewandten Arzneimitteln verschiedener Stoffklassen. So wird dieses Beschwerdebild häufig im Rahmen einer Chemotherapie sowie unter der Tumorbehandlung mit Aromatasehemmern oder Antiöstrogenen beobachtet. Auch hier können Veränderungen an der Vaginalhaut - wie Rötung und petechiale Blutungen – objektiv festgestellt werden. Das subjektive Beschwerdebild einer trockenen und empfindlichen Scheide und Vulva kann überdies auch ohne objektivierbaren Hintergrund vorkommen.

In der Regel wird bei postmenopausalen Frauen oder postoperativ bei Frauen nach Ovar-ektomie eine vaginale Östrogentherapie mit gutem Behandlungserfolg durchgeführt. Allerdings gibt es Situationen, in denen eine Östrogentherapie kontraindiziert oder nicht erwünscht ist.

Zur Verbesserung der Scheidenfeuchtigkeit, der Regeneration der Vaginalschleimhaut bzw. zur Reduktion der vaginalen Beschwerden sind verschiedene Hormon-freie Medizinprodukte im Handel erhältlich. Hierbei handelt es sich in der Regel um Produkte auf Gel-Basis. Bis zur Einführung des Hormon-freien Medizinproduktes Vagisan® FeuchtCreme in den Handel gab es keine Hormon-freie intravaginal anzuwendende Creme zur Behandlung der vaginalen Trockenheit bei Patientinnen mit dem beschriebenen Beschwerdebild.

In einer placebokontrollierten Doppelblindstudie mit Linoladiol® N Creme (Östradiolhaltige Creme zur Behandlung der vulvovaginalen Atrophie; Dr. August Wolff Arzneimittel) ließ sich im Hinblick auf die Besserung der subjektiven Beschwerden zwischen Verum und Placebo (wirkstofffreie Grundlage) kein statistisch signifikanter Unterschied feststellen. Dies war der Ausgangspunkt für die Entwicklung von Vagisan® FeuchtCreme, die sich lediglich durch den Zusatz eines Milchsäurepuffers und den Gehalt an Benzylalkohol (2% statt vorher 1%) von der Linoladiol N-Grundlage unterscheidet.

Der Zusatz des Milchsäurepuffers bewirkt in der Creme einen pH Wert von 4,5 und hat zum Ziel, einen physiologischen pH-Wert im Scheidenmilieu (der bei gesunden Frauen prämenopausal bei pH 4,0 – pH 4,4 liegt) zu erhalten bzw. wiederherzustellen.

Vagisan® FeuchtCreme (Dr. August Wolff Arzneimittel) ist ein im Handel befindliches, zertifiziertes Medizinprodukt der Klasse II b. Bei der Anwendung von Vagisan® FeuchtCreme handelt es sich um eine rein symptomatische Therapie, bei der der Haut in der Scheide und an der Vulva Feuchtigkeit und pflegende Lipide (Creme) zugeführt werden.

Bereits durchgeführte Untersuchungen

Präklinische Studien (pharmakologische, pharmakokinetische und toxikologische Studien) liegen für das wirkstofffreie Medizinprodukt Vagisan® FeuchtCreme nicht vor. Allerdings sind alle in Vagisan® FeuchtCreme eingesetzten Substanzen Bestandteil einer Vielzahl zugelassener vaginal anzuwendender Cremes (Arzneimittel) und daher vielfach präklinisch und klinisch untersucht.

Eine prospektive, multizentrische, kontrollierte, randomisierte, Medizinprodukt-Studie zur Untersuchung der Wirksamkeit und Verträglichkeit von Vagisan® FeuchtCreme (Prüf-Medizinprodukt) im Vergleich zu Gynomunal® Vaginalgel (Referenz-Medizinprodukt) im 2-Perioden-Change-over-Design an 120 Patientinnen mit trockener Scheide, die keine Östrogene anwenden wollten oder durften, wurde im Jahr 2008 durchgeführt. Im Vorfeld dieser Studie wurde eine Sichtung und kritische Wertung der relevanten wissenschaftlichen Literatur durchgeführt.

Für das Hauptzielkriterium “Besserung der subjektiven Beschwerden“ konnte statistisch gesichert (5% Niveau) eine Überlegenheit von Vagisan® FeuchtCreme im Vergleich zu Gynomunal® Vaginalgel nachgewiesen werden. Es ließ sich nicht nur eine Besserung der subjektiven Beschwerden, sondern auch des objektiven Befundes der vaginalen Trockenheit feststellten.

Nach der Anwendung von Vagisan® FeuchtCreme bzw. Gynomunal® Vaginalgel kam es häufig (mit einer errechneten Wahrscheinlichkeit von 0,04 bzw. 0,08) zu einem vorübergehenden Gefühl von Brennen und Jucken; bei je einer Patientin in jeder Behandlungsgruppe hielt diese Reaktion länger als 12 Stunden an und wurde als „Unverträglichkeit“ eingestuft. Im Gesamturteil zur Verträglichkeit der Präparate bewerteten sowohl die Patientinnen als auch die behandelnden Ärzte Vagisan® FeuchtCreme hochsignifikant besser als Gynomunal® Vaginalgel ($p < 0{,}01$).

Produktinformation

Bestandteile
Benzylalkohol, Cetylpalmitat, Cetylstearylalkohol, Milchsäure, Natriumlactat, Octyldodecanol, Polysorbat 60, Sorbitanstearat, gereinigtes Wasser. Vagisan FeuchtCreme ist frei von Duftstoffen.

Bei Vagisan® FeuchtCreme handelt es sich um eine weiße, geschmeidige Creme, die für die Anwendung bei dem Beschwerdebild der Scheidentrockenheit (vulvovaginale Trockenheit) entwickelt worden ist. Vagisan® FeuchtCreme ist frei von Hormonen (Östrogenen) und kann deshalb auch zusammen mit einer Hormonersatztherapie oder im Wechsel mit Hormon-(Östrogen-) haltigen Vaginal-Cremes oder Vaginalzäpfchen angewandt werden.

Durch den Zusatz von Milchsäure ist die Creme auf einen pH-Wert von 4,5 eingestellt – damit unterstützt Vagisan® FeuchtCreme die Erhaltung eines natürlichen pH-Wertes in der Scheide. Zwei Effekte tragen dazu bei, dass Vagisan® FeuchtCreme die Beschwerden bei Scheidentrockenheit lindert: Die Creme hat einen hohen Wassergehalt, dadurch wird der Haut im Bereich der Scheide und des äußeren Intimbereichs Feuchtigkeit zugeführt; pflegende Lipide halten die Haut geschmeidig.

Die regelmäßige Anwendung von Vagisan® FeuchtCreme kann dazu beitragen, Reizungen und Entzündungen in der Scheide und im äußeren Intimbereich vorzubeugen. Die Creme kann bei Scheidentrockenheit vor dem Geschlechtsverkehr angewandt werden, grundsätzlich auch zusammen mit Latex-Kondomen. Ist eine Schwangerschaft erwünscht, bestehen gegen die Anwendung von Vagisan® FeuchtCreme bei Scheidentrockenheit keine Einwände, da die Beweglichkeit der Spermien nicht beeinträchtigt wird. Eine Anwendung während der Menstruation ist möglich.

Anwendung von Vagisan FeuchtCreme

Die Creme ist zur Anwendung in der Scheide und auf der Haut des äußeren Intimbereichs bestimmt. Vagisan FeuchtCreme kann mit und ohne Applikator angewendet werden.

Bei Beschwerden im Scheideneingang und im äußeren Intimbereich wird die Creme nach Bedarf mehrfach täglich mit einem sauberen Finger aufgetragen und verteilt; dafür reicht ein Cremestrang von ca. 0,5 cm Länge (1 Fingertip-unit) aus. Eine geringe Menge Creme kann im Bereich des Scheideneingangs auch aufgetragen werden, um das Einführen des Applikators zu erleichtern.

Bei vaginalen Beschwerden wird Vagisan® FeuchtCreme mit Hilfe des beigefügten Applikators in die Scheide eingeführt, anfangs täglich, jeweils abends vor dem Schlafengehen. Dazu wird der Applikator bis zur Hälfte befüllt (ca. 2,5 g Creme). Nach Besserung der Beschwerden kann die Anwendungshäufigkeit nach Bedarf herabgesetzt werden.

Mögliche Nebenwirkungen

Gelegentlich können – aufgrund des Milchsäure-Zusatzes - vorübergehend leichte lokale Reizungen (Juckreiz, Brennen) und ggf. leichter Ausfluss vorkommen. Sehr selten können Überempfindlichkeitsreaktionen (z. B. Rötung, Brennen) auftreten.

Anwendungsbeschränkungen

Vagisan FeuchtCreme darf nicht angewendet werden bei bekannter Überempfindlichkeit oder Allergie gegenüber einem der Bestandteile. Bei Vorliegen einer Scheideninfektion ist Vagisan FeuchtCreme nicht anzuwenden.

<u>Nutzen-Risiko-Beurteilung</u>

Alle in Vagisan® FeuchtCreme eingesetzten Substanzen sind bekannt und finden in einer Vielzahl von zugelassenen Arzneimitteln Verwendung. Eine mit Vagisan® FeuchtCreme durchgeführte klinische Studie bei 92 Frauen mit dem Beschwerdebild der vulvovaginalen Trockenheit zeigte eine gute Wirksamkeit (i.S. einer Linderung der subjektiven Beschwerden und Besserung des objektiven Befundes) und Verträglichkeit.

Aufgrund des Milchsäure-Zusatzes kann es nach dem Auftragen zu einem vorübergehenden leichten Gefühl von Brennen oder Jucken kommen. Wie bei jedem auf Haut und Schleimhäuten anzuwendenden Produkt lässt sich – im Sinne eines potentiellen Risikos - nicht ausschließen, dass in individuellen Fällen eine lokale Unverträglichkeitsreaktion (auch im Sinne einer allergischen Reaktion) auftreten kann.

Eine Risikoanalyse nach den für Medizinprodukte gültigen Richtlinien wurde durchgeführt. Danach sind für das Medizinprodukt Vagisan® FeuchtCreme keine relevanten Gefährdungen identifiziert worden. Vagisan® FeuchtCreme ist als wirksames und sicheres Medizinprodukt zu bewerten. Der Nutzen des Produktes für die Anwenderin übersteigt deutlich das potentielle Risiko.

4. Ziel der Anwendungsuntersuchung

Ziel dieser Anwendungsuntersuchung ist die Beantwortung der Frage, ob die Anwendung von Vagisan® FeuchtCreme bei Brustkrebs-Patientinnen unter Chemotherapie bzw. Therapie mit Antiöstrogenen / Aromatasehemmern eine Besserung der subjektiven Beschwerden und des objektiven Befundes einer vulvovaginalen Trockenheit bei guter Verträglichkeit bewirken kann.

Vagisan® FeuchtCreme soll auf folgende Parameter untersucht werden:

<u>Verträglichkeit</u>

- Art und Häufigkeit von Nebenwirkungen (Unerwünschten Medizinprodukt-Wirkungen, UMW)
- Allgemeine Urteile zur Verträglichkeit (Arzt / Patientin)

<u>Wirksamkeit</u>

- Subjektive Beschwerden (Trockenheitsgefühl; Juckreiz; Brennen; Schmerzen unabhängig vom Geschlechtsverkehr; Schmerzen beim Geschlechtsverkehr; Harninkontinenz)
- Objektive Befunde (Verdünnung des Vaginalepithels; Rötung; petechiale Blutungen; Fluor)
- Allgemeine Urteile zur Wirksamkeit (Arzt / Patientin)

<u>Gesamtbewertung des Medizinproduktes</u>

- Zusammenfassende Beurteilung des Medizinproduktes Vagisan® FeuchtCreme (Arzt / Patientin)

5. Qualitätssicherung

Diese Anwendungsuntersuchung berücksichtigt soweit wie möglich die von der „Deutschen Gesellschaft für medizinische Informatik, Biometrie und Epidemiologie“ (GMDS) 1997 publizierten „Empfehlungen zur Durchführung von Anwendungsbeobachtungen“ sowie die vom Bundesinstitut für Arzneimittel und Medizinprodukte (BfArM) und dem Paul Ehrlich Institut (PEI) am 09. Mai 2007 bekannt gemachte Entwurfsfassung „Gemeinsame Empfehlungen zur Planung, Durchführung und Auswertung von Anwendungsbeobachtungen“.

Als besondere Maßnahmen zur Qualitätssicherung dienen im Vorfeld:

- Darstellung und Vermittlung der besonderen Bedeutung, die jede an einer Anwendungsuntersuchung beteiligte Person besitzt, um im Rahmen einer Anwendungsuntersuchung zu qualitativ hochwertigen Daten zu gelangen.

- Genaue Regelungen der Vorgehensweise für alle Schritte der Anwendungsuntersuchung, insbesondere auch bzgl. Aspekten zur Pharmakovigilanz.

Die Dokumentation der Patientinnendaten erfolgt online mittels elektronischer Dokumentationsbögen (eCRFs = electronic case report forms). Der Arzt hat auf eine vollständige Dokumentation zu achten. Die elektronisch erfassten Untersuchungsdaten werden durch das RDE-System automatisch auf Vollständigkeit und Plausibilität geprüft.

Unvollständige oder nicht plausible Angaben, die die Medizinproduktsicherheit berühren, werden von unmittelbar nach Kenntnisnahme direkt mit dem Arzt abgeklärt. Medizinproduktsicherheitsrelevante Vorkommnisse werden auf Basis von SUEs oder bewerteten unerwünschten Ereignissen (Definitionen SUE bzw. UE siehe Kap. 8) dokumentiert.

6. Beobachtungsumfang/Organisation

In der Zeit vom 04.01.2010 bis ca. 30.06.2010 sollen 150 prämenopausale Patientinnen ab 18 Jahren mit Mamma-Carcinom unter Chemotherapie ODER Therapie mit Antiöstrogenen oder Aromatasehemmern (aktuelle Therapie bzw. bis zu 3 Monate nach Beendigung der Therapie) UND mit dem Beschwerdebild der vulvovaginalen Trockenheit in die Anwendungsuntersuchung aufgenommen werden. Die Patientinnenzahl von 150 wurde für ausreichend angesehen, um ein valides Ergebnis zu erhalten hinsichtlich der Zielfrage, ob eine Besserung der subjektiven Beschwerden und des objektiven Befundes einer vulvovaginalen Trockenheit bei guter Verträglichkeit erreicht werden kann. Jede Patientin wird über 28 Tage mit Vagisan® FeuchtCreme behandelt.

Frauen, die bereits vor Beginn der Tumorbehandlung das Beschwerdebild der vulvovaginalen Trockenheit hatten, sollen nicht an der Anwendungsuntersuchung teilnehmen. Patientinnen mit anderen Tumoren als dem Mamma-Carcinom und Patientinnen, die eine Bestrahlungstherapie erhalten, sind von der Anwendungsuntersuchung ausgeschlossen. Erkrankungen an Vagina oder Vulva sowie eine aktuelle zusätzliche Therapie der vulvovaginalen Trockenheit oder vulvovaginalen Atrophie, sind weitere Ausschlusskriterien. Patientinnen mit bekannter Überempfindlichkeit gegenüber einem der Inhaltsstoffe von Vagisan® FeuchtCreme oder einer aktuell vorliegenden Vaginalinfektion dürfen nicht an der Anwendungsuntersuchung teilnehmen. Frauen, die nicht in der Lage sind die Studie ordnungsgemäß durchzuführen, oder an einem aktuellen Alkohol- oder Drogenabusus leiden, dürfen nicht in die Anwendungsuntersuchung eingeschlossen werden. Schwangere und stillende Frauen dürfen nicht an dieser Anwendungsuntersuchung teilnehmen.

Die Anwendungsuntersuchung wird multizentrisch, in gynäkologischen Praxen durchgeführt.

Das Medizinprodukt Vagisan® FeuchtCreme sowie die zur Anwendung erforderlichen Applikatoren werden den Ärzten vom Sponsor, Dr. August Wolff Arzneimittel, zur Verfügung gestellt.

Vor der Teilnahme eines Arztes an der Anwendungsuntersuchung muss die Teilnahmeerklärung des Arztes ausgefüllt und unterschrieben vorliegen. Vor der Aufnahme einer Patientin in die Studie muss dem Arzt nach entsprechender Aufklärung der Patientin, die Patientinneneinwilligung zur Teilnahme an der Anwendungsuntersuchung von der Patientin unterzeichnet vorliegen.

Die Unterlagen zur Anwendungsuntersuchung werden jedem teilnehmenden Arzt von der zur Verfügung gestellt. Die Dokumentation erfolgt online mittels elektronischer Dokumentationsbögen (eCRF = electronic case report form). Hierbei erhält der teilnehmende Arzt, nachdem die Teilnahmeerklärung unterschrieben wurde, von der einen Zugangscode, der den elektronischen Zugang zur Dokumentation ermöglicht. Die Online-Dokumentation soll möglichst zeitnah nach dem Einschluss einer Patientin in die Anwendungsuntersuchung erfolgen.

Der den Ärzten durch das Ausfüllen der elektronischen Dokumentationsbögen im Rahmen dieser Anwendungsuntersuchung entstehende Mehraufwand wird in Anlehnung an die GOÄ honoriert. Die vollständig ausgefüllten Dokumentationsbögen werden mit € (netto) je Patientin vergütet.

Durch diese Anwendungsuntersuchung wird weder Einfluss auf die Behandlungsempfehlungen oder Verordnungen des Arztes genommen noch die Therapiefreiheit eingeschränkt. Die Behandlung muss in Übereinstimmung mit der Gebrauchsanweisung von Vagisan® FeuchtCreme erfolgen und sollte den individuellen medizinisch therapeutischen Notwendigkeiten entsprechen.

7. Beobachtungsablauf

Bezüglich der Therapie wird ausdrücklich auf die Gebrauchsanweisung von Vagisan® FeuchtCreme verwiesen. Dieser sind alle wichtigen Informationen (wie z.B. Anwendungsgebiete, Nebenwirkungen, Wechselwirkungen, Gegenanzeigen, Art und Dauer der Anwendung, Dosierung) zu entnehmen.

Vor Beginn der Anwendungsuntersuchung wird die Patientin schriftlich und mündlich durch den teilnehmenden Arzt über den Ablauf der Anwendungsuntersuchung aufgeklärt. Jede potentielle Studienteilnehmerin hat vor ihrer Aufnahme ihr schriftliches Einverständnis zur freiwilligen Teilnahme an dieser Anwendungsuntersuchung zu erklären.
Anhand der Ein- und Ausschlusskriterien wird die Eignung der Patientin zur Teilnahme an dieser Anwendungsuntersuchung geprüft.
Jeder Patientin wird bei Aufnahme in die Anwendungsuntersuchung eine individuelle Patientinnennummer zugeteilt, mit der alle Dokumente der Patientin gekennzeichnet werden. Alle teilnehmenden Ärzte sind verpflichtet, die Teilnahme der Patientin an dieser Anwendungsuntersuchung in den Patientinnenunterlagen zu vermerken. Medizinische Informationen über die Patientin sollten nur anonymisiert weitergeleitet und analysiert werden. Die Identität aller Patientinnen, die an dieser Anwendungsuntersuchung teilnehmen, muss sorgfältig unter Verschluss gehalten werden. Im Falle von Nachfragen, besonders im Zusammenhang mit Nebenwirkungsmeldungen, muss der Arzt in der Lage sein, die Anonymisierung der in dieser Anwendungsuntersuchung beobachteten Patientin aufzuheben. Zu diesem Zweck erfolgt der Eintrag von Patientinnennummer und Name in die Patientinnenidentifikationsliste. Diese verbleibt in der Praxis und muss ebenfalls sorgfältig unter Verschluss gehalten werden.

Visite 1: Tag 0, vor Beginn der Anwendung von Vagisan® FeuchtCreme

1. Arzt
   - Gynäkologische Untersuchung

     Erhebung der objektiven Befunde: Verdünnung des Vaginalepithels, Rötung, petechiale Blutungen, Fluor; Bewertung des Schweregrades anhand einer Skala von 0 bis 4 (0 = keine, 1 = gering, 2 = mäßig, 3 = stark, 4 = sehr stark ausgeprägt)
   - Ärztliche Erfassung der malignen Erkrankung (Brustkrebs) und ggf. weiterer nicht-onkologischer Erkrankungen, der onkologischen Therapie und ggf. weiterer nicht-onkologischer Medikationen (aktuell)

2. Patientinnen-Fragebogen
   - Erhebung der subjektiven Beschwerden: Trockenheitsgefühl, Juckreiz, Brennen, Schmerzen unabhängig vom Geschlechtsverkehr, Schmerzen beim Geschlechtsverkehr; Bewertung des Schweregrades anhand einer Skala von 0 bis 4 (0 = keine, 1 = gering, 2 = mäßig, 3 = stark, 4 = sehr stark ausgeprägt), Harninkontinenz
   - Jeweils mit Angabe, seit wann die Beschwerden bestehen

- Angabe, ob bereits Präparate gegen Scheidentrockenheit angewendet wurden. Falls ja, Nennung des Präparates. Pauschale Bewertung der Wirksamkeit und Verträglichkeit (jeweils mit den „Noten“ 1-6) (1 = sehr gut bis 6 = ungenügend)

Tag 0 - 28: Anwendung von Vagisan® FeuchtCreme

Intravaginal:

1.Woche: täglich 1 x 2.5 g (eine halbe Applikatorfüllung) abends

2. – 4. Woche: täglich oder nach Bedarf seltener, 1 x 2.5 g abends

Im äußeren Intimbereich:

durchgehend 4 Wochen nach Bedarf, auch mehrfach täglich, jeweils ca. 0.5 g (1 Fingertip-unit).

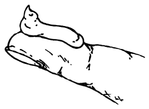

Visite 2: Tag 28, Abschluss der Untersuchung

1. Arzt

- Gynäkologische Abschlussuntersuchung mit Befundung (wie an Tag 0)
- Ärztliche Erfassung jeglicher weiterer Erkrankungen und Medikationen in den letzten 4 Wochen
- Erfassung bekannter Unerwünschter Medizinproduktwirkungen (UMW) von Vagisan® FeuchtCreme (Befragung der Patientin):
    - o Vorgegebene Parameter: „Vorübergehendes Jucken oder Brennen (unter 10 Minuten)“, „länger anhaltendes Jucken oder Brennen (10 bis 60 Minuten)“, „starke Unverträglichkeit (Jucken, Brennen, Schwellung länger als 60 Minuten)“, „Ausfluss der Creme“
    - o Befragung der Patientin nach SUEs und UEs, ärztliche Bewertung des Kausalzusammenhangs und im Fall positiver Kausalität (s. Abschnitt 8) Dokumentation als Nebenwirkung (Unerwünschte Medizinproduktwirkung, UMW)
- Ärztliche Beurteilung:
    - o Allgemeines Urteil zur Verträglichkeit („Noten“ 1-6)*
    - o Allgemeines Urteil zur Wirksamkeit („Noten“ 1-6)*
    - o Zusammenfassende Beurteilung des Medizinproduktes Vagisan® FeuchtCreme („Noten“ 1-6)*

      * 1 = sehr gut bis 6 = ungenügend

2. Patientinnen-Fragebogen

- Häufigkeit der intravaginalen Anwendung in der 2. bis 4. Woche
- Häufigkeit der Anwendung im äußeren Intimbereich
- Erhebung der subjektiven Beschwerden: Trockenheitsgefühl, Juckreiz, Brennen, Schmerzen unabhängig vom Geschlechtsverkehr, Schmerzen beim Geschlechtsverkehr; Bewertung des Schweregrades anhand einer Skala von 0 bis 4 (0 = keine, 1 = gering, 2 = mäßig, 3 = stark, 4 = sehr stark ausgeprägt), Inkontinenz
- Allgemeines Urteil zur Verträglichkeit („Noten“ 1-6)*
- Allgemeines Urteil zur Wirksamkeit („Noten“ 1-6)*
- Zusammenfassende Beurteilung des Medizinproduktes Vagisan® FeuchtCreme („Noten“ 1-6)*

* 1 = sehr gut bis 6 = ungenügend

8. Definitionen zur Medizinproduktsicherheit

Unerwünschte Ereignisse[1]

Ein **unerwünschtes Ereignis** ist jedes schädliche Ereignis, das einem Patienten nach Verabreichung eines Medizinproduktes widerfährt, unabhängig davon, ob ein kausaler Zusammenhang mit dieser Behandlung vermutet worden ist oder ob ein bestimmungsgemäßer Gebrauch vorliegt.

Unerwünschte Medizinproduktwirkung (UMW)[1]

Unerwünschte Medizinproduktwirkung ist hier synonym zu verstehen zu den Begriffen „Nebenwirkung“ und „Verdachtsfall einer unerwünschten Medizinproduktwirkung“. Nebenwirkungen sind schädliche, unbeabsichtigte Reaktionen, die beim bestimmungsgemäßen Gebrauch eines Medizinproduktes auftreten.

Eine unerwünschte Medizinproduktwirkung ist im Gegensatz zu einem unerwünschten Ereignis dadurch charakterisiert, dass zwischen verabreichtem Medizinprodukt und dem beobachteten Ereignis der Verdacht eines Kausalzusammenhangs besteht, d.h. der Zusammenhang muss vom behandelnden Arzt mindestens als „unwahrscheinlich“ eingestuft sein.

Schwerwiegende Nebenwirkungen / schwerwiegende unerwünschte Ereignisse[1] **/ Vorkommnisse**

Schwerwiegende Nebenwirkungen / unerwünschte Ereignisse (SUEs; engl.: serious adverse events = SAEs) sind solche, die tödlich oder lebensbedrohend sind, eine stationäre Behandlung oder Verlängerung einer stationären Behandlung erforderlich machen, zu bleibender oder schwerwiegender Behinderung, Invalidität, kongenitalen Anomalien oder Geburtsfehlern führen oder solche, die medizinisch bedeutsam sind.

Schwerwiegende Nebenwirkungen sind synonym zu dem Begriff „Vorkommnisse“ (gem. Medizinproduktesicherheitsverordnung) zu verstehen und beinhalten eine unerwünschte Medizinproduktwirkung, die unmittelbar oder mittelbar zum Tod oder zu einer schwerwiegenden Verschlechterung des Gesundheitszustands eines Patienten, eines Anwenders oder einer anderen Person geführt hat, geführt haben könnte oder führen könnte.

Medizinisch bedeutsame Nebenwirkungen / unerwünschte Ereignisse sind solche, die nicht sofort tödlich oder lebensbedrohlich sind oder zu einer stationären Behandlung führen, aber den Patienten erheblich beeinträchtigen oder die eine Intervention / Behandlung zur Verhinderung eines Zustandes erfordern, der der Definition für „schwerwiegend“ entspricht. (z.B. Ereignisse, die Maßnahmen erfordern, die eine Krankenhauseinweisung verhindern.)

---

[1] - Definitionen gemäß § 4 Abs. 13 Arzneimittelgesetz (AMG) und „5. Bekanntmachung zur Anzeige von Nebenwirkungen und Arzneimittelmissbrauch nach § 63b Abs. 1 bis 8 des Arzneimittelgesetzes“ vom 05.12.2007.

Bei bekannt oder vermutlich teratogenen Präparaten ist auch eine Schwangerschaft ein schwerwiegendes unerwünschtes Ereignis.
Eine ungeplante Schwangerschaft erfüllt immer die Kriterien eines schwerwiegenden unerwünschten Ereignisses.

In der Regel zählt als stationäre Behandlung ein Krankenhausaufenthalt, der eine Übernachtung einschließt. Stationäre Behandlungen aus rein diagnostischen Gründen, routinemäßigen Kontrolluntersuchungen oder bei bereits vor Beginn der Anwendungsuntersuchung geplanten operativen Eingriffen und aus sozialer Indikation stellen keine Hospitalisierung im Sinne des Begriffes „schwerwiegend“ dar.

Bewertung des Kausalzusammenhangs

(Nach WHO-UMC Causality Categories [UMC = Uppsala Monitoring Centre])

Kausalzusammenhang „gesichert“ (UMC – certain)
Ein klinisches Ereignis – einschließlich anomaler Laborbefunde – das in einem plausiblen zeitlichen Zusammenhang mit der Verabreichung eines Medizinproduktes auftritt und nicht durch eine Begleiterkrankung oder andere Arzneimittel bzw. Chemikalien erklärt werden kann. Die Reaktion nach Absetzen des Medizinproduktes (Dechallenge) sollte klinisch plausibel sein. Das Ereignis muss pharmakologisch oder phänomenologisch eindeutig sein, wofür ggf. ein geeignetes Reexpositionsverfahren (Rechallenge) verwendet werden sollte.

Kausalzusammenhang „wahrscheinlich“ (UMC – probable/likely)
Ein klinisches Ereignis – einschließlich anomaler Laborbefunde – das in einem angemessenen zeitlichen Zusammenhang mit der Verabreichung eines Medizinproduktes auftritt und wahrscheinlich nicht auf eine Begleiterkrankung oder andere Arzneimittel bzw. Chemikalien zurückzuführen ist, wobei das Absetzen des Medizinproduktes (Dechallenge) eine klinisch plausible Reaktion hervorruft. Angaben über eine Reexposition sind nicht erforderlich, um diese Definition zu erfüllen.

Kausalzusammenhang „möglich“ (UMC – possible)
Ein klinisches Ereignis – einschließlich anomaler Laborbefunde – das in einem angemessenen zeitlichen Zusammenhang mit der Verabreichung eines Medizinproduktes auftritt, aber auch durch eine Begleiterkrankung oder andere Arzneimittel bzw. Chemikalien erklärt werden könnte. Angaben über die Reaktion nach Absetzen des Medizinproduktes können fehlen bzw. unklar sein.

Kausalzusammenhang „unwahrscheinlich“ (UMC – unlikely)
Ein klinisches Ereignis – einschließlich anomaler Laborbefunde – das in einem zeitlichen Zusammenhang mit der Verabreichung eines Medizinproduktes steht, wobei aufgrund des Zeitraumes ein ursächlicher Zusammenhang unwahrscheinlich ist und andere Arzneimittel, Chemikalien oder Grundkrankheiten plausible Erklärungen darstellen.

Kausalzusammenhang „kein Zusammenhang“
Ausgeschlossen als Verdachtsfall einer Nebenwirkung sind alle unbeabsichtigten Reaktionen, die evident andere – innere oder äußere – Ursachen als die Gabe des Medizinproduktes haben. Dazu gehören auch Symptome, die eindeutig Ausdruck der Grunderkrankung oder Begleiterkrankung des Patienten sind. Hier sollte bei Verschlimmerung der Krankheitssymptome auf die Möglichkeit einer Ähnlichkeit von Krankheitssymptomatik und Nebenwirkung sorgfältig geprüft werden, ob eine medizinproduktbedingte Nebenwirkung vorliegt und ob im Zweifel eine Anzeige erstattet wird.

Ausgeschlossen als Verdachtsfälle von Nebenwirkungen sind auch Symptome, die nachweislich in gleicher (oder stärkerer) Intensität vor der Verabreichung des Medizinproduktes auftraten oder solche, bei denen sich herausgestellt hat, dass das genannte Medizinprodukt nachweislich nicht angewendet wurde.

Kausalzusammenhang „nicht zu beurteilen“ (UMC – conditional/unclassified)
Ein klinisches Ereignis – einschließlich anomaler Laborbefunde – das als unerwünschte Medizinproduktwirkung berichtet wurde, für das mehr Daten notwendig sind für eine angemessene Bewertung, oder die zusätzlichen Daten werden zur Zeit geprüft.

Zusätzlich wird die folgende Definition unter „nicht zu beurteilen“ eingeschlossen
(UMC – unassessible/unclassifiable):
Ein Bericht, der eine unerwünschte Medizinproduktwirkung nahe legt, die nicht beurteilt werden kann, da die zur Verfügung stehenden Informationen nicht ausreichend oder widersprüchlich sind, und die nicht ergänzt oder überprüft werden können.

9. Meldung bzw. Dokumentation von SUE, Unerwünschten Medizinproduktwirkungen (UMW) sowie Verdachtsfällen von UMW

Da es sich bei dieser Anwendungsuntersuchung um eine Dokumentation im Rahmen des üblichen Anwendungsgebietes des zertifizierten Medizinproduktes Vagisan® FeuchtCreme handelt, werden nur „Schwerwiegende Unerwünschte Ereignisse" (Schwerwiegende Nebenwirkungen / schwerwiegende unerwünschte Ereignisse[1] / Vorkommnisse; zur Definition siehe Kapitel 8), Unerwünschte Medizinproduktwirkungen sowie Verdachtsfälle von Medizinproduktnebenwirkungen dokumentiert. Der Kausalzusammenhang eines „Unerwünschten Ereignisses“ mit der Medikation muss vom Arzt also zumindest als „unwahrscheinlich“ beurteilt werden (genaue Definitionen siehe Kapitel 8), um ein „Unerwünschtes Ereignis“ zu einer „UMW“ (Unerwünschte Medizinproduktwirkung) werden zu lassen. Explizit werden Ereignisse, die nicht schwerwiegend sind und die erkennbar keinen Kausalzusammenhang mit der Medikation aufweisen, nicht gesondert erfasst.

Unerwünschte Medizinproduktwirkungen (UMW) sowie Verdachtsfälle von UMW (Kausalität ärztlicherseits mindestens als „unwahrscheinlich“ beurteilt) werden im Rahmen der 2. Visite zum Abschluss der Studie erfasst und dokumentiert.

Schwerwiegende unerwünschte Ereignisse (Schwerwiegende Nebenwirkungen / schwerwiegende unerwünschte Ereignisse[1] / Vorkommnisse; Definition s. Kapitel 8), die im Rahmen der Anwendungsuntersuchung auftreten, sind umgehend nach Kenntnisnahme zu dokumentieren und innerhalb von 24 Stunden vom Arzt an weiterzuleiten:

Zur Erfassung stehen die Formblätter „Meldung von schwerwiegenden unerwünschten Ereignissen (SUEs)“ und „Bericht über Verdachtsfälle unerwünschter Medizinproduktwirkungen (UMW)“ zur Verfügung.

Falls im Verlauf der Anwendungsuntersuchung ein SUE auftritt, das den Abbruch der Anwendungsuntersuchung bedingt, wird dies vom Arzt dokumentiert und innerhalb von 24 Stunden nach Kenntnis an weitergeleitet. Von sind SUEs sofort nach Kenntnisnahme an den Sponsor weiterzuleiten.

10. Data Management und Biometrische Auswertung

Im Verlauf der Studie erfolgt die Befunddokumentation rechnergestützt mittels einer Online Remote Data Entry (RDE) Applikation, die analog dem von der entwickelten Beobachtungsbogen (CRF) von der erstellt wird. Die Zugriffs-berechtigung zu diesem Online-Datenerfassungssystem wird über Zugangscodes geregelt. Eintragungen und Korrekturen dürfen nur vom teilnehmenden Arzt und seinen dazu autorisierten Mitarbeitern vorgenommen werden. Korrekturen werden so erfasst, dass der alte Eintrag weiterhin abgerufen werden kann. Alle Eingaben und Korrekturen werden mit Datum, Uhrzeit und Namen der eintragenden Person automatisch protokolliert ("Audit trail").

Die rechnergestützte Datenerfassung durch den Arzt erfolgt plausibilitätsgeprüft, entspricht den Anforderungen der FDA und der EMEA und erfüllt höchste Qualitätsstandards.

Zwecks Identifizierung der Patientin ist vom teilnehmenden Arzt eine Patientinnenidentifikationsliste zu führen. Die Teilnahme an der Anwendungs-untersuchung (Patientennummer/Beginn und Ende der Teilnahme/Medizinprodukt) ist ebenfalls in der Patientenakte des Arztes zu vermerken.

In dieser Anwendungsuntersuchung wird eine deskriptive Statistik eingesetzt.

Auswertung folgender Parameter:

- Art und Häufigkeit von SUEs
- Art und Häufigkeit von Nebenwirkungen (Unerwünschte Medizinproduktwirkungen sowie Verdachtsfälle auf UMW)
- Allgemeine Urteile zur Verträglichkeit (Arzt / Patientin)

- Subjektiver Beschwerde-Score (Einzelscores, Summenscore; jeweils Änderung Tag 0 minus Tag 28)
- Objektiver Befund-Score (Einzelscores, Summenscore; jeweils Änderung Tag 0 minus Tag 28)
- Allgemeine Urteile zur Wirksamkeit (Arzt / Patientin)
- Zusammenfassende Beurteilung des Medizinproduktes Vagisan® FeuchtCreme (Arzt / Patientin)

- Anamnestische Angaben der Patientin zur vulvovaginalen Trockenheit (Dauer, Vorbehandlung), Harninkontinenz
- Häufigkeit der Anwendung von Vagisan® FeuchtCreme, intravaginal und im äußeren Intimbereich
- Tumorerkrankung (Diagnose) und ggf. weiterer Erkrankungen (anamnestisch und im Verlauf der Studie)

- Art der Tumortherapie und ggf. weiterer Medikationen (anamnestisch und im Verlauf der Studie)

11. Anzeigepflicht

Vor Beginn der Anwendungsuntersuchung soll das Votum der Freiburger Ethi[illegible] Kommission International eingeholt werden.

Da es sich bei dieser multizentrischen Anwendungsuntersuchung nicht um eine klinische Prüfung gem. MPG und gem. Richtlinie 93-42-EWG handelt, besteht keine Anzeigepflicht bei einer Behörde. Dennoch ist es vorgesehen, die für klinische Prüfungen mit Medizinprodukten zuständige Landesbehörde über die Durchführung der Anwendungsuntersuchung zu informieren. Die Mitteilung umfasst die Meldung der Anwendungsuntersuchung sowie die namentliche Meldung der teilnehmenden Ärzte. Da es sich bei Vagisan® FeuchtCreme um ein nicht verordnungsfähiges und nicht erstattungsfähiges Medizinprodukt handelt und die erforderlichen Präparate den Teilnehmerinnen der Untersuchung durch den Sponsor zur Verfügung gestellt werden, ist eine Anzeige der Anwendungsuntersuchung bei der kassenärztlichen Bundesvereinigung und den Spitzenverbänden der Krankenkassen nicht vorgesehen.

Nach Abschluss der Anwendungsuntersuchung wird ein ausführlicher Bericht erstellt, der eine statistische Auswertung sowie eine Bewertung der Ergebnisse aus medizinischer Sicht umfasst. Die Richtigkeit des Inhalts wird durch die Unterschriften des Leiters der Anwendungsuntersuchung, des Projektleiters beim Sponsor, des für die statistische Auswertung verantwortlichen Biometrikers und des für die Berichterstellung verantwortlichen Mitarbeiters der bestätigt.

12. Literatur

1. Mazur D; Vens-Cappell B; Lohmann K; Breckwoldt M: Fraktionierte Anwendung einer 17beta Estradiolcreme zur Behandlung der atrophischen Kolpitis postmenopausaler Frauen. *Geburtshilfe und Frauenheilkunde; 65/6, 584-589 (2005)*

2. SOGC Clinical Practice Guidelines: The detection and management of vaginal atrophy International. *Journal of Gynecology and Obstetrics; 88/2, 222-228 (2005)*

3. Bachmann GA, Nevadunsky NS: Diagnosis and Treatment of Atrophic Vaginitis. *American Family Physician. 2000 May 15;61(10):3090-6*

4. Mok K, Juraskova I, Friedlander M: The impact of aromatase inhibitors on sexual functioning: Current knowledge and future research directions. *The Breast;17, 436-440 (2008)*

5. Bruno D, Feeney KJ: Management of postmenopausal symptoms in breast cancer survivors. *Semin Oncol; 33:696-707 (2006)*

6. Dr. August Wolff GmbH & Co. KG Arzneimittel: Vagisan® FeuchtCreme – Gebrauchsanweisung. *Stand der Information: März 2009*

7. Dr. August Wolff GmbH & Co. KG Arzneimittel: Vagisan® FeuchtCreme - Studie zur Wirksamkeit und Verträglichkeit. *Synopse des Studienberichts, Dr. August Wolff (2008)*

13. Unterschriften

Leiter der Anwendungs-
untersuchung: ________________________ ____________

Datum

Projektleiter beim Sponsor: ________________________ ____________

Datum

Fachreferat Gynäkologie,
beim Sponsor: ________________________ ____________

Datum

Leiterin Monitoring bei : ________________________ ____________

Datum

Biometriker und Projektverant-
wortlicher bei : ________________________ ____________

Datum
